# Community-based participatory design of a decade: the FAITH! Cardiovascular Health and Wellness Program

**DOI:** 10.3389/fpubh.2025.1622237

**Published:** 2025-09-23

**Authors:** LaPrincess C. Brewer, Mathias Lalika, Ashley N. Kyalwazi, Monica Albertie, Janice Bowie, Ashya Burgess, Lora E. Burke, Brian Buta, Lisa A. Cooper, Deidra C. Crews, Chyke A. Doubeni, Walé Elegbede, Jamia Erickson, Sarah Jenkins, Jacquelyn Johnson, Clarence Jones, Ashton Krogman, Lainey Moen, Michael Palmer, Christi A. Patten, Sumedha Penheiter, Monisha W. Richard, Princess Titus, Sueling Schardin, Stanton Shanedling, Jeremy R. Van’t Hof, David Warner, Jennifer Weis, Sharonne N. Hayes

**Affiliations:** 1 Department of Cardiovascular Medicine, Mayo Clinic, Rochester, MN, United States; 2 Center for Clinical and Translational Science, Mayo Clinic, Rochester, MN, United States; 3 San Francisco Department of Medicine, University of California, San Francisco, San Francisco, CA, United States; 4 Center for Clinical and Translational Science, Mayo Clinic, Jacksonville, FL, United States; 5 Johns Hopkins Bloomberg, School of Public Health, Baltimore, MD, United States; 6 Division of Hematology, Comprehensive Cancer Center, Mayo Clinic, Rochester, MN, United States; 7 Department of Health and Community Systems, School of Nursing, University of Pittsburgh, Pittsburgh, PA, United States; 8 Division of Geriatric Medicine and Gerontology, Center on Aging and Health, Johns Hopkins University, Baltimore, MD, United States; 9 Department of Medicine, Johns Hopkins University, School of Medicine, Baltimore, MD, United States; 10 Department of Family and Community Medicine, Wexner Medical Center, The Ohio State University, Columbus, OH, United States; 11 Strategy Management Services, Mayo Clinic, Rochester, MN, United States; 12 National Association for the Advancement of Colored People, Rochester, MN, United States; 13 Thrivent Financial, Incorporated, Rochester, MN, United States; 14 Department of Quantitative Health Sciences, Mayo Clinic, Rochester, MN, United States; 15 Christ’s Church of the Jesus Hour, Rochester, MN, United States; 16 Hue-Man Partnership, Minneapolis, MN, United States; 17 Revival Home Health and Hospice, Baltimore, MD, United States; 18 Department of Psychiatry and Psychology, Mayo Clinic College of Medicine, Rochester, MN, United States; 19 Strategy Operations, Mayo Clinic, Rochester, MN, United States; 20 The Linc, Minneapolis, MN, United States; 21 Appetite For Change, Minneapolis, MN, United States; 22 American Heart Association, Eagan, MN, United States; 23 Heart Disease and Stroke Prevention Unit, Minnesota Department of Health, Saint Paul, MN, United States; 24 Cardiovascular Division and Lillehei Heart Institute, University of Minnesota Medical School, Minneapolis, MN, United States; 25 Department of Anesthesiology and Perioperative Medicine, Mayo Clinic, Rochester, MN, United States

**Keywords:** cardiovascular health, community-based participatory research, health disparities, health equity, social determinants of health

## Abstract

The FAITH! (Fostering African-American Improvement in Total Health) Cardiovascular Health and Wellness Program is more than a decade-long community-based participatory research initiative aimed at addressing cardiovascular health disparities among African-Americans in Minnesota. Founded in 2013, the program employs a culturally tailored, community-driven approach by partnering with African-American faith communities to promote cardiovascular health through education, digital health tools, and multilevel interventions targeting the social determinants of health. Grounded in community-based participatory research principles, FAITH! prioritizes equitable academic-community partnerships, co-learning, community capacity building, and shared ownership in all aspects of research and implementation. The program’s exemplary innovations include the NIH-funded FAITH! Trial, a randomized clinical trial, testing a mobile health intervention (the FAITH! App) co-created with the African-American community, and the Techquity by FAITH! study. Techquity by FAITH! evaluates the effectiveness of a culturally relevant, community-informed mHealth intervention supported by a Digital Health Advocate network to improve overall cardiovascular health and digital health literacy. During its evolution, FAITH! has addressed emergent public health crises, including the COVID-19 pandemic, by adapting programming to provide emergency preparedness resources, health education, and vaccine outreach. Key outcomes include sustainable church-based health ministries, increased research participation, and successful translation of research into practice. The program has also contributed to research workforce development by mentoring and training diverse early-career scholars and community leaders in community-based participatory research and cardiovascular health equity research. Lessons learned highlight the transformative impact of community-based participatory research in building trust, facilitating culturally relevant dissemination, and sustaining health equity initiatives. The FAITH! model demonstrates a scalable, community-led strategy for advancing cardiovascular health in underserved populations and provides a blueprint for future initiatives aiming to reduce racial health disparities.

## Introduction

In 2023, the Fostering African-American Improvement in Total Health (FAITH!) Cardiovascular Health (CVH) and Wellness Program celebrated 10 years of successful health promotion among African-Americans in Minnesota. This success was not without challenges, unexpected setbacks and new directions heralded by the unprecedented COVID-19 public health crisis. Nonetheless, the FAITH! founding director and Principal Investigator, Dr. LaPrincess Brewer, a preventive cardiologist and community activist, was guided by committed community partners to hold to the FAITH! Program’s founding mantra to “*take care of your body and your spirit.*” In doing so, this team aimed to tackle disparities in cardiovascular disease (CVD) through promotion of healthy lifestyle and behavior change, which has led to more than a decade of sustainability.

The FAITH! Program began in 2008 with the early success of a classroom-based chronic disease prevention program spearheaded by Dr. Brewer and peer public health students within New Friendship Baptist Church, an African-American church in East Baltimore, Maryland, near Johns Hopkins Hospital (1). Despite the church’s over 30-year history in Baltimore and location in the “backyard” of Johns Hopkins, New Friendship was never approached for health interventions development partnerships with the prestigious hospital. Dr. Brewer and her fellow public health student classmates worked closely with Rev. Dr. Michael Palmer, their New Friendship community partner, to promote CVH through nutritional education within the church community. By applying the principles of community-based participatory research (CBPR), recognizing the faith community as a unit of identity, this project demonstrates that integrating culture, community values, and health education can promote better lifestyle choices among participants and foster a sustainable “culture of health.” This initial partnership with New Friendship served as a catalyst for larger scale interventions and programming to promote ideal CVH and tackle disparities in CVD among African-Americans in the state of Minnesota.

## Context

### The (not so) good life in Minnesota: FAITH! origins in Minnesota

Minnesota, the “Land of 10,000 Lakes,” is home to over 5 million individuals and has been consistently recognized for being one of the healthiest states in the United States with glowing perceptions of “The Good Life” reflecting quality of life and ample economic opportunity (2). However, the disproportionate burden of CVD incidence and mortality among African-American individuals in comparison to White individuals living in Minnesota tells a much different and disheartening narrative. African-Americans aged 35–63 years in Minnesota are more than twice as likely to die from CVD than White adults of the same age, in part because of higher rates of CVD risk factors such as diabetes, obesity, and physical inactivity (3). These CVD disparities “Up South” stem from historical contexts of African-American migration from the Deep South to northern regions in optimistic pursuit of opportunities for economic upward mobility (4). Despite migration to Northern states, African-Americans continue to experience high poverty rates, chronic stress from racism and inequality, and health outcomes similar to or worse than those in the South (3, 4).

In 2012, Dr. Brewer relocated to Rochester, Minnesota, for clinical fellowship training in Cardiology at the Mayo Clinic (Mayo) flagship medical center. She quickly recognized the striking racial disparities in CVH, accentuated marginalization of African-Americans in the region, and the potential for integrating FAITH! into medically underserved and socioeconomically disenfranchised communities throughout Minnesota to address systemic health inequities. Addressing these disparities would require a CBPR approach, which is core to the FAITH! Program. This includes community-based interventions, trust-building through shared decision-making, culturally tailored approaches, and equitable, evidence-based strategies to reduce the burden of preventable diseases and improve CVH in African-American populations.

## Programmatic elements: applying the CBPR principles

CBPR is a research paradigm which emphasizes community-centric strategies for culturally relevant programs to advance health equity (5). CBPR is an equitable, strength-based approach involving all stakeholders throughout the research process. Table 1 summarizes the efforts of FAITH! and its partners to operationalize CBPR principles (6).

**Table 1:** Operationalization of the community-based participatory research (CBPR) principles by the FAITH! program.

CBPR principles	Exemplary operationalization
Recognizes community as a unit of identity.	Recognized African-American faith community as a cultural unit and actively built a meaningful, sustained relationship with this group. Valued African-American community norms, customs, values, and social needs when conceptualizing research.
Builds on strengths and resources within the community.	Established the FAITH! Community Steering Committee (CSC), an advisory board learning body of community leaders and academic researchers to provide guidance through the entire research process. Partnered with trusted community members (e.g., community health workers and church liaisons) for study recruitment and health promotion, leveraging their lived experiences to enhance program outreach. In acknowledgement as a strong community-academic partnership, FAITH! CSC members selected to attend CBPR Partnership Academy and Engage for Equity Partnership Evaluation Workshop for hands-on, real-world professional development to further strengthen FAITH! programming.
Facilitates collaborative partnerships in all phases of the research.	FAITH! leadership earned trust and built relationships with local African-American community and addressed community-identified needs (e.g., nutrition education) to establish a community-academic partnership. Co-designed all research studies (e.g., CVD Prevention Program Pilot, *FAITH! Trial*, *Techquity by FAITH!* study, etc.) with African-American churches, ensuring shared decision-making and centering community priorities, values and culture.
Integrates knowledge and action for the mutual benefit of all partners.	Hosts annual Red Dress/Red Tie Sunday events in local African-American churches to increase awareness of cardiovascular disease in African-American women. Addresses pressing community health needs through skill-building to promote actionable research and community benefit (e.g., CPR training, blood pressure measurement, American Heart Association Life’s Essential 8).
Promotes a co-learning and empowering process that attends to social inequalities.	Community members receive training on academic research process; ensure academic team integrating health equity metrics.
Involves a cyclical and iterative process	FAITH! CSC engages in annual retreats to evaluate partnership effectiveness, assess quality of FAITH! programming, review lessons learned, brainstorm community-driven solutions and discuss strategic partnerships. Established local and national partnerships with local and national organizations (e.g., Balm in Gilead, Inc., Association of Black Cardiologists, Inc.). All FAITH!-partnering churches sustain independently operated health ministries. Mentoring of research trainees in CBPR at various career stages (e.g., medical students, postdoctoral fellows, etc.)
Addresses health from both positive and ecological perspectives.	Engaged with community members for participatory, user-centered design of a community-informed mHealth intervention (the *FAITH! App*). Repurposed to focus on COVID-19 pandemic (FAITH! Emergency Preparedness Initiative, COVID-19 testing site, COVID-19 vaccine townhall). Co-developed the FAITH! Heart Health+ study to address adverse social determinants of health and stressors (e.g., death of Mr. George Floyd) exacerbating CVH inequities during the pandemic.
Disseminates findings and knowledge gained to all partners.	Relays study findings back to participants and community at-large through public events. Community partners participate in conferences and professional society meetings and co-present with academic partners on study findings. Strategically partners with local and national media outlets to increase awareness about FAITH! initiatives.

### Principle 1: Recognizing the community as a unit of identity

With a history spanning over five centuries, the “Black Church” is a sacred cultural and societal unit, serving as a haven for religious worship but also a crucible for social support, political activism, education, health/social services, cultural expression and identity (7, 8). Recognizing this crucial role of the Black Church in the lives of many African-Americans, FAITH! intentionally partnered with these institutions from its founding. At the heart of the Black Church are African-American communities which have common norms, customs, and values as well as health and social needs. This also requires an appreciation that the African-American community is not monolithic and a more nuanced understanding of geographical, social and historical contexts is warranted when conceptualizing research (9). FAITH! recognized the multifaceted, distinctive communities by integrating cultural considerations in research, including the co-design of a culturally tailored mobile health lifestyle intervention (the *FAITH! App*) (10–15) and a Digital Health Equity toolkit for an ongoing study to improve digital literacy among African-Americans (16).

### Principle 2: Building on community strengths and resources

One of the core strategies of FAITH! in advancing health equity in African-American communities is to leverage existing community resources and strengths. The FAITH! Community Steering Committee (CSC) was established in 2017 using a rigorous, theory-informed, implementation process with the objective to better engage with the community as true collaborators and partners (17). This 20-member body is comprised of a diverse unification of academic researchers, faith leaders, community strategists, public health representatives, patient advocates, community health workers (CHWs), and other community leaders, which “steers” or guides the program’s activities to ensure that they are community-centered and actionable. Diversity in the group is a vital resource to understanding the myriad of community priorities from multiple perspectives, thus guiding both the studies and community interventions. More importantly, by representing the voices of the communities they belong to and providing valuable insights as a form of “social capital,” this committee plays a key role in building academic-community trust and fostering greater community collaboration. In addition, the CSC serves to identify existing assets (such as health and social programs) and resources (e.g., culturally congruent healthy lifestyle coaches, CHWs, venues, etc.) instrumental to facilitating team-based and implementation science (18) components within all FAITH! projects.

Furthermore, the FAITH! study recruitment efforts are primarily led by African-American community members (e.g., FAITH! Partners, CHWs, etc.), who leverage their lived experiences to promote our studies within their communities and networks. Similarly, these community partners utilize their community mobilization skills (e.g., appropriate locations, timing, activities, etc.) to inform the planning of community events to maximize engagement and participation. Acknowledging the accomplishments of our CBPR partnership, CSC co-leaders (L. B., C. J.) and a CSC member (M. W.) were invited to participate in the Engage for Equity (19) Partnership Evaluation Workshop at the University of New Mexico in January 2019, which convened prominent CBPR partnerships from across the United States for a collaborative learning experience (20, 21).

### Principle 3: Facilitating collaborative and equitable partnerships

The foundational activities of FAITH! emphasized community trust-building and equitable partnership. Upon the relocation of Dr. Brewer to Mayo, she approached the Mayo Center for Clinical and Translational Science (CCaTS) to leverage existing community-engaged initiatives and partnerships between Mayo and the surrounding African-American community. At that time there were limited collaborations in place between Mayo and the surrounding African-American community and health disparities research was in its early development. Dr. Brewer initiated a series of activities with select African-American church leaders in Rochester to gauge the interest of pastors in launching FAITH! in Rochester.

First, FAITH! held “listening and transparent communication” meetings with pastors and auxiliary leaders within each church for relationship-building and to gain understanding of the needs and priorities of the African-American community in Rochester (22). The response was mixed: some community leaders were enthusiastic to build a program focused on this historically marginalized African-American population, while others expressed outright disinterest as the program was perceived as a “hit and run” research project wrought by traditional academic power structures.

Dr. Brewer stepped back from the research focus to better understand the concerns of the congregation members. It became apparent that this skepticism stemmed, in part, from limited prior interaction with Mayo, including few opportunities for sustained and meaningful engagement with the African-American community in Rochester. There was also a clear misunderstanding of the research process, particularly surrounding informed consent. Further, reasonable privacy concerns about research data collection and how this information would be used to benefit individuals and the greater good of the community were also expressed. There was a shared sentiment among community members questioning the decision to frame the project as research, as opposed to positioning it as a community-oriented initiative reflecting Mayo’s commitment to giving back. Further meetings were held with community “gatekeepers” within each church and local African-American organizations [e.g., National Association for the Advancement of Colored People (NAACP) leadership] for additional understanding of community assets.

Dr. Brewer and her team also attended church-sponsored worship services and community outreach programming. The team’s initiative of continuing to “show up” helped earn trust and allowed them to become more trustworthy to a community that was marginalized within a city centered around a medical center considered as a global “powerhouse.” With a historical context of distrust in medical research stemming from atrocities involving the federal government [e.g., Tuskegee Study of Syphilis in the Negro Male (23) sponsored by the US Public Health Service] and large medical institutions [e.g., Henrietta Lacks cell line at Johns Hopkins (24, 25)], the FAITH! team prioritized transparent and consistent communication with church congregations, highlighting how this program and its rigorous, community-centric approach would ultimately benefit the community.

In addition, several listening sessions between Mayo team leaders and church members helped foster mutual understanding and strengthen connections between the institution and surrounding African-American communities, which had historically limited engagement with Mayo’s facilities, research, and innovation (26). Notably, some participants shared that, despite living nearby for decades, they had never visited the Mayo campus and had chosen to receive care elsewhere.

To demonstrate its commitment to the community and FAITH!, Mayo partnered with FAITH! to address a community-identified need for nutrition education and healthy food preparation. FAITH! collaborated with the Mayo Dan Abraham Healthy Living Center to host a live cooking demonstration in a state-of-the-art kitchen as a means to acknowledge and respond to the community’s priorities. The culinary team prepared traditional African-American cuisine using healthier and plant-based ingredients to improve the nutritional content. This intentional approach of genuinely welcoming the community into the “ivory tower” was well received and appreciated by the community. Although somewhat of a reversal of “meeting people where they are” in the community, it was a clear display of cultural humility and trust-building.

**Figure 1:**
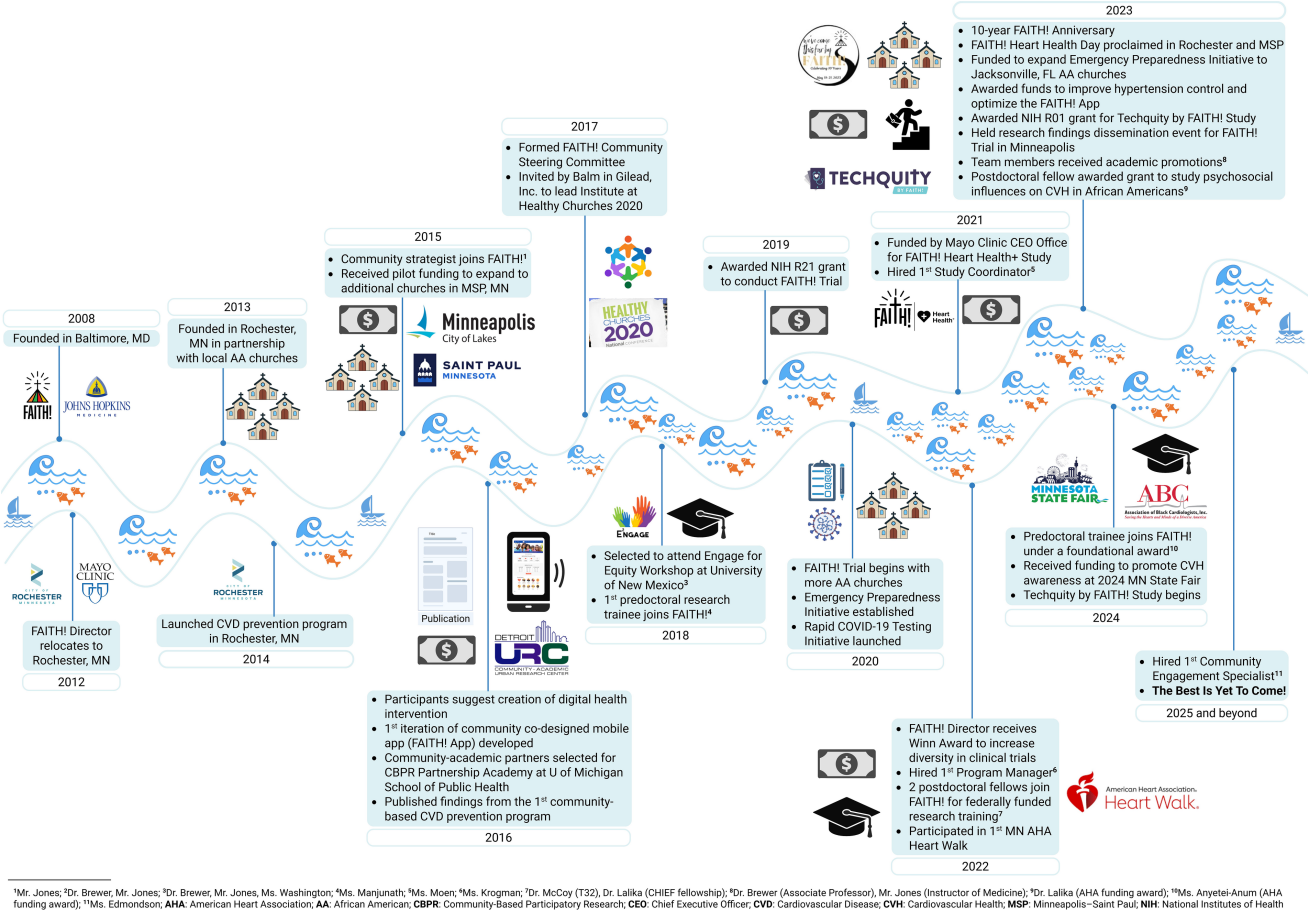
River of life of the FAITH! (Fostering African-American Improvement in Total Health!) program activities and milestones. A historical timeline of selected community-based participatory research activities and milestones achieved by FAITH! since its initial founding in Maryland and expansion to the FAITH! Cardiovascular Health and Wellness Program in Minnesota.

In 2013, upon establishing a mutual understanding of shared goals and using the CBPR tenets of engagement, several churches in Rochester requested to have FAITH!’s programming as a continued part of their health ministries with a new focus on CVD prevention to address health disparities most affecting their congregations and surrounding communities (22). As an equity-first, power-sharing model, each church identified a church liaison, designated as a FAITH! Partner, to serve as a connector for the church in all research decision-making processes. This marked the official establishment of FAITH! in Rochester. Similar strategies, employing lessons from this process, were used to expand our network of church partners in the Minneapolis-St. Paul metropolitan area. This collaborative formative stage of genuine partnership laid the groundwork for all future research, community-based interventions and outreach implemented by FAITH!. The African-American faith community engaged equally to develop research questions, design studies, recruit participants, collect data, interpret results, and disseminate findings. Figure 1 illustrates a historical timeline of selected CBPR activities and milestones achieved by FAITH! since its founding, using the River of Life (27), a reflective tool that outlines the history of a CBPR partnership and highlights how the progression has influenced the current state of the partnership.

### Principle 4: Integrating knowledge and action for mutual benefit

Consistent with its overarching goal of improving CVH among African-Americans as identified and mutually agreed upon by community members, FAITH!’s comprehensive suite of interventions focuses on CVH promotion and CVD prevention. For instance, the rigorously tested *FAITH! App* is currently being optimized into a smartphone-based application to expand dissemination to other African-American communities beyond Minnesota. In addition, insights from the FAITH! Trial (28) are being utilized to develop not only a commercially viable app but also a tool with advanced technical capabilities to address barriers to research participation among African-Americans. By enabling remote data collection, the app addresses challenges such as unreliable transportation and scheduling conflicts, thus facilitating participation in decentralized clinical trials (29).

Beyond research, FAITH! engages in initiatives to drive social change and raise awareness about community-identified challenges. Our research and dissemination events are complemented by non-research activities, including live cooking demonstrations and fitness classes, such as Zumba and Afro Karibe, to cultivate a community culture that promotes engagement in healthy behaviors. In 2018, the program collaborated with a local YMCA and church community to organize the “Walk by FAITH!” celebration series. This included a heart health fair, a health education presentation by Dr. Brewer, 2-mile walks in Rochester and Minneapolis, and a Men’s Health Symposium (30). In recognition of American Heart Month, FAITH! also hosts annual Red Dress/Red Tie Sunday events (31) within its church networks to increase awareness of CVD in African-American women.

FAITH! also leverages high-profile events to address pressing issues within the African-American community. For example, following NFL athlete, Mr. Damar Hamlin’s public cardiac arrest in 2023, FAITH!, collaborated with the local American Heart Association (AHA) affiliate to sponsor community-wide cardiopulmonary resuscitation and automated external defibrillator training at the Mayo Dan Abraham Healthy Living Center (32, 33). Additionally, FAITH! and its affiliated churches participate annually in the AHA Heart Walk to promote CVH awareness. To further engage with its community partners, FAITH! distributes a quarterly newsletter featuring resources such as internships, scholarships, podcasts, and upcoming seminars (Supplementary document 1). FAITH! also partners with national professional societies, such as the Association of Black Cardiologists, Incorporated (ABC), which has been instrumental in advancing community health advocacy (34) as well as multiple health policy changes and legislature at the federal level, such as Health Inequity and Diversity in Cardiology (35), Access to Care, and Tobacco Control (36).

### Principle 5: Promoting co-learning and community empowerment

The founding and expansion of FAITH! has been driven by a bidirectional co-learning process involving academic researchers and community partners/stakeholders. Central to FAITH! is its academic-community co-led study team, which includes the FAITH! founder and principal investigator (L. B.) and a former community engagement director of a federally qualified health center (FQHC) and community strategist and leader of a grassroots organization, Hue-Man Partnership in Minneapolis, Minnesota (C. J.). They were connected in 2015 through the director of the Minnesota Department of Health CVH Unit in St. Paul, Minnesota (S. S.), acknowledging their shared passions in CVD prevention in underserved communities. Seeking avenues to nurture the partnership, the co-leaders were one of 12 academic-community dyads selected to participate in the Detroit Community-Academic Urban Research Center CBPR Partnership Academy in July 2016 (37, 38). The Academy offered a dynamic co-learning environment that integrated formal coursework, hands-on CBPR training, professional development opportunities, and real-world experience to further FAITH!’s mission. The Academy also provided the co-leaders the impetus to form the FAITH! CSC (17). Through applied practice of concepts learned, the FAITH! academic partners learned strategies to authentically and effectively identify community needs through a deeper understanding of the culture and broader social context of the African-American community. This led to studies and interventions designed to effectively address the pressing health needs of the community.

Community partners have also gained insights into complex institutional cultures and processes of academic medical centers by participating in all stages of the research process. As a result, community partners have attained research knowledge and skills by actively partnering in study design, implementation, and dissemination, while holding the research team accountable for integration of health equity metrics. As an exemplar, our community partner co-leader (C. J.) has acquired knowledge, skills, and academic productivity, resulting in successful competitive research awards as a co-investigator and co-author on manuscripts and presentations at scientific meetings (39). Further, he received academic rank as an Instructor of Medicine at Mayo Clinic, a true demonstration of recognition for his contributions to the success of FAITH! and its benefits to the community. In addition, our academic team has prioritized having training sessions with community members to improve their skills for CVH assessment and promotion [e.g., blood pressure measurement, AHA Life’s Essential 8 (40) knowledge] within their communities.

### Principle 6: Cyclical and iterative process for long-term sustainability

FAITH! exemplifies the cyclical and iterative nature of CBPR through its sustained partnerships, co-developed interventions, collaborative research process, and commitment to long-term impact and sustainability (41). Through the FAITH! CSC and ongoing capacity-building efforts, the program addresses immediate health needs while empowering local African-American communities to improve long-term health outcomes. For example, through a cyclical and iterative learning process with embedded feedback mechanisms, CSC members participate in an annual retreat to: (1) evaluate partnership effectiveness in addressing health inequities (42), (2) assess FAITH! programming quality, (3) review lessons from FAITH! research advocacy and community outreach, (4) brainstorm community-driven solutions, and (5) discuss strategic partnerships to advance FAITH!’s mission.

For over 10 years, FAITH! demonstrated its commitment to sustainability through its collaborations with African-American churches in Rochester and Minneapolis-St. Paul, community-based organizations (e.g., Hue-Man Partnership, Appetite for Change), national professional societies and foundations (e.g., ABC, AHA, Robert Wood Johnson Foundation) and international faith-based organizations (e.g., The Balm In Gilead, Inc.). By leveraging academic and community resources and earning trust with African-American churches and the communities they serve, FAITH! has built a “from the ground up,” community-owned model for addressing health disparities in this population. All partnering churches now independently sustain health ministries that originated through FAITH!, with many continuing to utilize The Balm in Gilead’s *Sunday Morning Health Corner* (43) materials for health promotion. In Baltimore, FAITH! facilitated a post-worship service food pantry that continued to operate with sustained community support beyond the duration of the initial program (1).

FAITH! has also demonstrated sustainability through strategic funding, capacity building, and training future community-engaged researchers. Initially funded by Mayo CCaTS pilot grants, the program advanced to securing competitive federal and foundational funding from agencies, including the Centers for Disease Control and Prevention, the National Institutes of Health (NIH)/National Institute on Minority Health and Health Disparities (NIMHD), AHA and Robert Wood Johnson Foundation. One of its largest funding sources was an R01-equivalent supplement awarded in 2023 through the University of Minnesota-Mayo Clinic NIMHD P50 collaborative center grant, Center for Chronic Disease Reduction and Equity Promotion Across Minnesota (C2DREAM). The C2DREAM-funded *Techquity by FAITH!* study (44) aims to improve digital health literacy and CVH among African-Americans by co-designing a Digital Health Equity toolkit, training Digital Health Advocates with the ABC, and engaging them in a clinical trial to assess a community-informed mHealth intervention to address the digital divide.

To support CBPR capacity building, FAITH! actively trains researchers across career stages, including postbaccalaureate scholars, medical students, and postdoctoral fellows, through prestigious programs such as the Mayo-funded Cardiovascular Health Innovations in Equity (CHIEF) Fellowship and the NIH T32 Postdoctoral Fellowship. Trainees gain hands-on experience through integration into FAITH! activities and intensive CBPR coursework. FAITH! has also been designated as a community outreach opportunity within the Mayo graduate medical training programs (i.e., medical school, Internal Medicine residency, Cardiology fellowship programs). FAITH! trainees have secured competitive research awards (e.g., the AHA Research Supplement to Promote Diversity in Science), presented at national and international conferences, published in high-impact journals, and obtained positions within academia, government and industry (45–52).

### Principle 7: Addressing health through social determinants and ecological perspectives

Health disparities in Minnesota are driven by adverse social determinants of health (SDOH) (53), such as lack of home ownership, lower education levels, and income gaps. According to the 2022 Minnesota Poverty Report, poverty rates among African-Americans were nearly three times those of White individuals (54). To address these disparities, FAITH! focuses on CVD prevention in African-Americans through innovative, tailored interventions (e.g., smartphone apps, community webinars, forums, fitness classes, and cooking demonstrations) that “meet people where they are.” FAITH! tailors its studies to better understand and address the effects of socioeconomic disenfranchisement and systemic inequities on CVH, including identifying social needs (e.g., food and housing), stressors (55), sociocultural norms (56–59), and connecting community members to resources through a personalized, web-based referral platform (60).

Originating from community-generated ideas to develop a digital CVH platform, FAITH! conducted iterative pilot studies (11, 22, 61) that informed an NIH/NIMHD-funded, randomized clinical trial, the FAITH! Trial (62). This study tested the efficacy of the community co-created *FAITH! App* in improving CVH behaviors and clinical factors among African-American individuals (28, 62). The intervention integrated relevant psychosocial influences on CVH and offered practical, culturally appropriate solutions to promote CVH, including social support from peers and community leaders (13, 63–65).

During the COVID-19 pandemic, SDOH inequities (e.g., limited healthcare access) contributed to high infection, hospitalization, and mortality rates among African-Americans in Minnesota (66). In response, FAITH! pivoted and repurposed its research initiatives, with funding from CCaTS and the ABC, to address urgent community needs. In 2020, FAITH! used a CBPR approach to co-design “When Disaster Strikes: Keep Calm and Be Prepared,” an emergency preparedness (EP) initiative for the African-American community in Rochester and Minneapolis-St. Paul (67–69). Informed by a SDOH-based needs assessment, the initiative included a faith-based COVID-19 EP manual, a weekly “FAITH! & COVID-19 Spread the Word!” e-newsletter (Supplementary document 2), and a culturally-tailored social marketing campaign (including the ESSENCE Wellhouse Virtual Summit; Supplementary document 3) (70, 71). Led entirely by the FAITH! CSC COVID-19 Community Task Force, this initiative reached over 100 African-American churches and an estimated 12,000 individuals (now expanded to Jacksonville, Florida churches).

FAITH! also partnered with an FQHC in St. Paul to establish a COVID-19 prevention program and drive-through SARS-CoV-2 specimen collection site in a medically underserved community, when testing sites were largely limited to more affluent areas and home-based testing was unavailable (72). In 10 weeks, over 2,000 community members, including low-income individuals and essential workers, received health education and were tested. FAITH! further studied factors influencing COVID-19 vaccine acceptance among economically marginalized patients in Minnesota (73) as well as pandemic-related, psychosocial hardships among church congregations. To address vaccine hesitancy beyond Minnesota, FAITH! and Mayo Clinic leadership at all three sites (Minnesota, Arizona, Florida) organized a virtual Town Hall to provide comprehensive education on COVID-19 vaccine options (74–76). This event featured Dr. Kizzmekia Corbett, a key NIH researcher in the Moderna vaccine development, and Dr. Pernessa Seele, an immunologist, public health activist, and CEO of The Balm In Gilead, Inc. The event was well-attended, with over 200,000 streams within its first days of airing.

Coinciding with pandemic-related health disparities was the murder of Mr. George Floyd, an African-American man, by police in Minneapolis, which contributed to widespread grief, stress, and adverse mental health challenges among African-Americans (77). Recognizing the compounded impact of the syndemic of the COVID-19 pandemic (78), the death of Floyd, and the resultant civic unrest, FAITH! initiated an ancillary study within an existing randomized clinical trial (62), the FAITH! Heart Health+ Study (79, 92). The study received financial support as part of the greater Mayo anti-racism initiatives and Chief Executive Office’s clinical trials stimulus grants to support community-engaged research. The study investigated the influence of biopsychosocial factors on CVH among African-American Minnesotans previously enrolled in the FAITH! Trial. Preliminary findings from this study show that depressive symptoms, proximity to social unrest, and high effort coping are associated with worse CVH.

Together, these initiatives underscore FAITH!’s holistic social-ecological model and multisector approach to health promotion and research that acknowledges the dynamic and complex interplay between individuals and their immediate familial, social, communal, and broader societal context. This approach has bolstered long-term resilience and preparedness in the face of adversity in the African-American community.

### Principle 8: Disseminating findings and knowledge collaboratively

FAITH!’s research is community-centered, prioritizing the translation of findings to benefit the communities that the program serves. In this way, FAITH! is committed to ensuring research outcomes are effectively communicated back to the community. This is achieved through collaborative dissemination efforts, including in-person and virtual events, workshops, and seminars. Beyond publishing findings in peer-reviewed journals, the program organizes annual events to present results in accessible, lay-friendly language. Moreover, abstracts and manuscripts produced by the FAITH! team are co-authored with community partners, who actively contribute to the manuscript preparation process. Community partners also participate in national conferences and professional society meetings (e.g., AHA, American Public Health Association, Academy Health) alongside the academic team that has allowed them a venue to present study findings and engage in discussions with the global research community. Since 2015, FAITH! and Mayo have sponsored community partners to attend and present at the annual Balm in Gilead, Inc. Healthy Churches 2030 conference, including a Mayo-led workshop on enhancing representation of African-Americans in medical research which centered on the FAITH! model of community engagement (80, 81). Furthermore, FAITH! research milestones have received extensive coverage by local and national media outlets (including the oldest Black-owned newspaper in Minnesota, the Minnesota Spokesman Recorder and the Star Tribune) (82–87). This coverage has provided exceptional exposure to increase awareness about FAITH! initiatives and build a positive reputation within the community. In addition, FAITH! maintains an online presence through official social media platforms [e.g., Facebook (88) and X [formerly Twitter] (89)] and a dedicated website (90) to expand its capacity to engage with and disseminate health information to the diverse African-American community.

## Discussion

### Lessons learned and implications for future sustainability

In its first decade of implementation in Minnesota, FAITH! has gathered significant insights to inform its future work and long-term sustainability. In 2023, FAITH! celebrated 10 years of successful research and community engagement in Minnesota by hosting a weekend of curated events with our partnering churches, collaborators and supporters including a gala and a Sunday worship service. As a commemorative honor, May 20th was declared as FAITH! Heart Health Day in perpetuity by formal proclamations from the mayors of the cities of Rochester, Minneapolis, and St. Paul. This was truly an extraordinary moment for FAITH! and an inflection point of what is yet to come in the future. Since that time, our academic-community partner team and FAITH! collaborative network reflected on its key lessons learned as detailed below.

*Building Sustainable Academic-Community Partnerships*. Establishing trust requires time, cultural understanding, and academic humility. The iterative process of relationship-building emphasizes consistency and responsiveness to evolving community needs. Addressing pressing community challenges beyond health issues strengthens trust, incentivizes community participation in initiatives and fosters a shared commitment to addressing disparities. Through this partnership, FAITH! and its community partners have co-developed a mission to support actionable research aimed at improving community health, with the ultimate goal of effectively addressing CVH disparities in Minnesota, particularly within African-American communities.

*Transformative Power of CBPR Principles*. When applied effectively, CBPR principles enable meaningful research by enhancing trust and bridging the divide between health institutions and historically marginalized communities. For example, FAITH!’s longstanding collaboration with African-American church communities contributes to enhanced and trustworthy relationships between Mayo and the local African-American population, as evidenced by increased community engagement and institutional support for community-driven initiatives.

*Community-Driven Research Contributions*. Community members, when consistently engaged, actively inform every phase of the research process, from question formulation to data interpretation and dissemination of results (91). This participatory approach enhances research relevance and methodological rigor while empowering community members through their direct involvement.

*Community-Informed Dissemination*. Dissemination activities, such as workshops and seminars, are not merely endpoints but serve as opportunities to gather community input for refining subsequent research and interventions. For instance, feedback directly garnered from dissemination events informed the conception and development of the *FAITH! App*, which is now one of FAITH!’s signature interventions.

*Community Enthusiasm for Research Participation*. African-American communities are eager to engage in research that addresses their concerns and honor them as active collaborators in the process. Sustained engagement and transparent communication of findings are critical to fostering and maintaining this interest. For example, during FAITH! dissemination events, community members who had not previously participated in studies expressed interest in joining ongoing and future research efforts, highlighting the role of trust and perceived research value in encouraging participation.

*Leveraging Community Resources*. Communities possess inherent resources and expertise that can enhance research outcomes. By building trust, community members may openly share strategies to improve recruitment, identify key collaborators, and avoid missteps that could erode trust. For example, consultations with community partners shaped critical revisions to a digital health toolkit central to the ongoing Techquity by FAITH! randomized clinical trial (16).

*Ownership as a Sustainability Driver*. Long-term sustainability relies on fostering community ownership of research initiatives. When communities perceive research as a tool to address their health needs, their commitment drives the program’s longevity. FAITH!’s approach of addressing community-identified health priorities exemplifies this principle, creating a model that communities can sustain and expand.

*Replicating Best Practices for Expansion*. Lessons learned in Baltimore and Rochester were successfully adapted for expansion into Minneapolis and St. Paul, demonstrating the scalability of FAITH!’s model. For instance, the EP initiative co-developed by FAITH! with Minnesotan African-American communities during the COVID-19 pandemic now informs public health efforts in other regions of the United States (e.g., Jacksonville, Florida; Jackson, Mississippi; New Orleans, Louisiana).

*Necessity of Inclusive Research Practices*. Excluding community voices from research risks irrelevance, flawed methodologies, limited impact, and eroded trust. By engaging diverse community stakeholders through CBPR, FAITH! ensures that research questions and findings are relevant and actionable, thus increasing the likelihood of policy translation and systemic change.

These lessons underscore the importance of authentic partnerships, community engagement, and cultural humility in creating sustainable, impactful programs. Continuously integrating these principles positions FAITH! to expand and deepen its reach and impact on health disparities among African-Americans. Specifically, FAITH! continues to embed its interventions within existing community structures such as church-based health ministries, many of which now operate independently using tools and curricula introduced by the program. The *FAITH! App* is currently undergoing optimization for broader dissemination and commercialization, expanding its reach beyond Minnesota to other African-American communities across the United States. Additionally, the Digital Health Equity toolkit and Digital Health Advocate training model from the Techquity by FAITH! study are designed to be adaptable for other racial/ethnic populations and geographic contexts. By maintaining a flexible, culturally responsive framework grounded in CBPR principles, FAITH! remains well-positioned to be replicated in diverse settings, leveraging local partnerships and resources to address CVH disparities nationwide.

### Implementation challenges and adaptive strategies of the FAITH! program

Despite the successes of FAITH!, several challenges emerged throughout its implementation that warrant discussion. Participant recruitment and retention were ongoing obstacles, particularly during the COVID-19 pandemic (28, 60, 62). As public health restrictions limited in-person engagement, recruitment shifted to virtual platforms (28), which reduced opportunities for direct relationship-building, a cornerstone of our community-based participatory approach. Retention was also affected by pandemic-related stressors (60), competing life demands, and digital access barriers, especially among individuals with limited technological proficiency. Integration of community feedback, while foundational to our model, required significant time and resources to ensure that input was meaningfully incorporated into intervention design and implementation. For example, virtual focus groups facilitated continued community involvement during the pandemic, but attendance variability among participants occasionally hindered consistent engagement (65). Moreover, the urgency to address emergent community needs, such as vaccine outreach and emergency preparedness, sometimes delayed research timelines and constrained the scope of real-time intervention refinements. To address these barriers, the program employed flexible enrollment strategies, provided individualized technical assistance, and engaged trusted CHWs and church liaisons to serve as digital health navigators. While these efforts helped mitigate participation challenges, they also highlighted important limitations to scaling the intervention. Moving forward, future studies should prioritize enhanced recruitment infrastructure, hybrid engagement models that combine virtual and in-person components, and directed investments in digital equity. These experiences emphasize the necessity of maintaining adaptable, multimodal engagement approaches and underscore the critical role of digital infrastructure in supporting both the sustainability and scalability of community-based interventions, particularly in the context of public health crises and ongoing structural inequities.

## Conclusion

While much work remains to close racial gaps in the CVD burden for African-Americans living in Minnesota, FAITH! provides a compelling model of intentional and authentic deployment of robust, CBPR-focused approaches that are centered on community-identified needs and preferences to address health disparities. Over the last decade, FAITH! has expanded its community partners to include a network of committed churches, stakeholders, and organizations in the state of Minnesota. It has successfully designed and rigorously tested novel, community-driven interventions, establishing a solid evidence base for improving CVH and overall wellness among African-Americans. Ongoing and future efforts will remain steadfast in balancing community stewardship and scholarship as a surefire path to a lasting impact and to advance health equity.

## Data Availability

The original contributions presented in the study are included in the article/Supplementary material, further inquiries can be directed to the corresponding author.
