# Correction: Community-based participatory design of a decade: the FAITH! Cardiovascular Health and Wellness Program

**DOI:** 10.3389/fpubh.2026.1782256

**Published:** 2026-01-28

**Authors:** LaPrincess C. Brewer, Mathias Lalika, Ashley N. Kyalwazi, Monica Albertie, Janice Bowie, Ashya Burgess, Lora E. Burke, Brian Buta, Lisa A. Cooper, Deidra C. Crews, Chyke A. Doubeni, Walé Elegbede, Jamia Erickson, Sarah Jenkins, Jacquelyn Johnson, Clarence Jones, Ashton Krogman, Lainey Moen, Michael Palmer, Christi A. Patten, Sumedha Penheiter, Monisha W. Richard, Princess Titus, Sueling Schardin, Stanton Shanedling, Jeremy R. Van't Hof, David Warner, Jennifer Weis, Sharonne N. Hayes

**Affiliations:** 1 Department of Cardiovascular Medicine, Mayo Clinic, Rochester, MN, United States; 2 Center for Clinical and Translational Science, Mayo Clinic, Rochester, MN, United States; 3 San Francisco Department of Medicine, University of California, San Francisco, San Francisco, CA, United States; 4 Center for Clinical and Translational Science, Mayo Clinic, Jacksonville, FL, United States; 5 Johns Hopkins Bloomberg, School of Public Health, Baltimore, MD, United States; 6 Division of Hematology, Comprehensive Cancer Center, Mayo Clinic, Rochester, MN, United States; 7 Department of Health and Community Systems, School of Nursing, University of Pittsburgh, Pittsburgh, PA, United States; 8 Division of Geriatric Medicine and Gerontology, Center on Aging and Health, Johns Hopkins University, Baltimore, MD, United States; 9 Department of Medicine, Johns Hopkins University, School of Medicine, Baltimore, MD, United States; 10 Department of Family and Community Medicine, Wexner Medical Center, The Ohio State University, Columbus, OH, United States; 11 Strategy Management Services, Mayo Clinic, Rochester, MN, United States; 12 National Association for the Advancement of Colored People, Rochester, MN, United States; 13 Thrivent Financial, Incorporated, Rochester, MN, United States; 14 Department of Quantitative Health Sciences, Mayo Clinic, Rochester, MN, United States; 15 Christ's Church of the Jesus Hour, Rochester, MN, United States; 16 Hue-Man Partnership, Minneapolis, MN, United States; 17 Revival Home Health and Hospice, Baltimore, MD, United States; 18 Department of Psychiatry and Psychology, Mayo Clinic College of Medicine, Rochester, MN, United States; 19 Strategy Operations, Mayo Clinic, Rochester, MN, United States; 20 The Linc, Minneapolis, MN, United States; 21 Appetite for Change, Minneapolis, MN, United States; 22 American Heart Association, Eagan, MN, United States; 23 Heart Disease and Stroke Prevention Unit, Minnesota Department of Health, Saint Paul, MN, United States; 24 Cardiovascular Division and Lillehei Heart Institute, University of Minnesota Medical School, Minneapolis, MN, United States; 25 Department of Anesthesiology and Perioperative Medicine, Mayo Clinic, Rochester, MN, United States

**Keywords:** cardiovascular health, community-based participatory research, health disparities, health equity, social determinants of health

An incorrect **Funding** statement was provided with various funders erroneously omitted.

The correct **Funding** statement reads:

The author(s) declared that financial support was received for this work and/or its publication. The research reported herein was supported by: the Association of Black Cardiologists, Inc., the Clinical and Translational Science Awards (CTSA) from the National Center for Advancing Translational Sciences (NCATS) to Mayo Clinic (Grant No. UL1 TR000135), the Florida Heart Research Foundation/Miami Heart Research Institute, the Johns Hopkins University Alumni Association, the Johns Hopkins Urban Health Institute, the Mayo Clinic Center for Health Equity and Community Engagement in Research, the Mayo Clinic Chief Executive Office, and the Mayo Clinic Office of Digital Innovation and the National Institutes of Health (NIH)/National Institute on Minority Health and Health Disparities (NIMHD) (Grant No. 1 R21 MD013490–01). Brewer was supported by the American Heart Association/Robert Wood Johnson Foundation-Amos Medical Faculty Development Program (Grant No. 19AMFDP35040005), Bristol Myers Squibb Foundation, Centers for Disease Control and Prevention (CDC, Grant No. CDC-DP18-1817), American Heart Association Second Century Implementation Science Award (Grant No. 23SCISA1144689), KL2 Mentored Career Development Program (NIH/CTSA Grant No. KL2 TR002379), NIH Building Interdisciplinary Research Careers in Women's Health (BIRCWH) Scholar Award (Grant No. K12 HD065987), and NIH/NIMHD P50 Center for Chronic Disease Reduction and Equity Promotion Across Minnesota (C2DREAM) Program (Grant No. P50MD017342) during the implementation of this work. Lalika received funding from the American Heart Association Research Supplement to Promote Diversity in Science Award (Grant No. 23DIVSUP1067167) and the NIH/NIMHD P50 C2DREAM Program (Grant No. P50MD017342) during the implementation of this work. The content of this manuscript is solely the responsibility of the authors and do not necessarily represent the official views of the CDC, NCATS, or the NIH. The funders had no role in study design, data collection and analysis, decision to publish, or preparation of the manuscript.

The original version of this article has been updated.

