# Supplementary material for: Community-based participatory design of a decade: the FAITH! Cardiovascular Health and Wellness Program
Source: Front Public Health. 2025 Sep 23;13:1622237. doi: 10.3389/fpubh.2025.1622237 (PMC12500619; doi:10.3389/fpubh.2025.1622237)
Supplement: Supplementary file 1 [file Data_Sheet_1.PDF]

**SUPPLEMENTAL MATERIAL**

# Community-Based Participatory Design of a Decade: The FAITH! Cardiovascular Health and Wellness Program

This supplement contains the following elements:

**Supplemental Document 1**. FAITH! Quarterly Newsletter

**Supplemental Document 2**. FAITH! & COVID-19 Spread the Word!" e-newsletter

**Supplemental Document 3**. 10 Commandments for a Healthy Heart During the COVID-19 Pandemic Flyer

# We've Come This Far By FAITH! Newsletter

## Awards/Grants

*With support and guidance from the FAITH! team and Community Steering Committee, Dr. Brewer has received the following awards recognizing her contribution to the public health and cardiology fields through her work with FAITH! and her dedication to the health of her patients and community:*

**"Techquity by FAITH" project granted funding by The Center for Chronic Disease Reduction and Equity Promotion Across Minnesota (C2DREAM)**

We are excited to announce that the FAITH! Program has been awarded a 2.2M, 3-year Supplemental Grant from The Center for Chronic Disease Reduction and Equity Promotion Across Minnesota (C2Dream) for our "Techquity by FAITH!" project. Click here to read more.

We are in the process of planning kick-off events. Please keep an eye out for more information.

**"FAITH! App" project granted funding by Mayo Clinic**

We are also pleased to share we have been awarded a $70k, 1-year Innovation Accelerator Award from Mayo Clinic Office of Digital Innovation for our "FAITH! App" project.

## FAITH! in the News

*Dr. Brewer, the FAITH! Program and collaborators have been featured in news stories and media outlets throughout the year.*

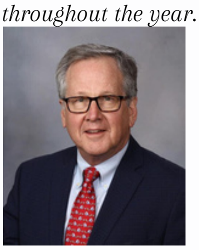

As a part of the Minnesota Cardiovascular Health Alliance, this group recently released **Minnesota's Action Plan to Address Cardiovascular Disease, Stroke, and Diabetes 2035** and focused on health equity.

This initiative was led by our very own Dr. Stephen Kopecky, Mayo Clinic Cardiologist.

Click here to review how Minnesota is creating conditions where all Minnesotans can thrive.

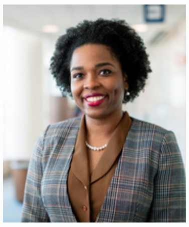

Our work was highlighted in the **American Heart Association Scientific Statement** on the Implementation of Prevention Science to Eliminate Health Care Inequities. This statement was featured in CIRCULATION.

Dr. Brewer contributed to the recently published **American Heart Association Presidential Advisory** on Food Is Medicine as a co-author. The article was published in CIRCULATION. Please feel free to review and share the news release about the article.

## Presentations/Conferences

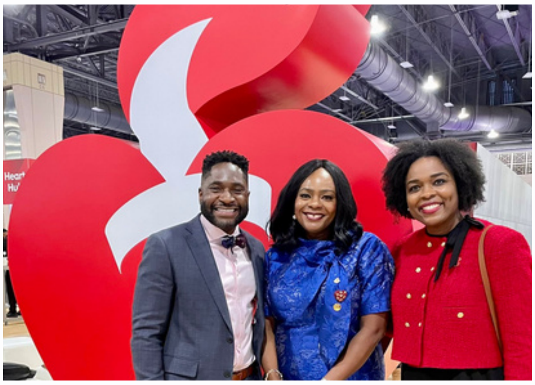

*Pictured left to right: Dr. Utibe Essien, Dr. Michelle Albert (immediate-past American Heart Association President) and Dr. Brewer at the AHA Awardee Reception.*

In November 2023, Dr. Brewer attended the **2023 American Heart Association Scientific Meeting** in Philadelphia, PA. Dr. Brewer's highly anticipated presentation: "Implementing Life's Essential 8 in Clinical Practice" was well-received and a conference favorite. Dr. Brewer also moderated two sessions: "Healthcare Informatics and Health Equity: Opportunities and Challenges", and "AI in the Clinic and Lab: Promise, Pitfalls, and the Data Police".

## WHAT'S INSIDE

Awards/Grants
FAITH! in the News
Presentations/Conferences
Recent Publications
Recent Outreach Events
Brewer Collaboratory News
Career, Training & Funding Oppor.
Upcoming Events

## Recent Publications about FAITH!, Heart Health, and Public Health

**Role of Sex in the Association of Socioeconomic Status With Cardiovascular Health in Black Americans: The Jackson Heart Study**
Published in Journal of the American Heart Association on December 1, 2023. You can access the article here.

**Efficacy and Adherence Rates of a Novel Community-Informed Virtual World-Based Cardiac Rehabilitation Program: Protocol for the Destination Cardiac Rehab Randomized Controlled Trial**
Published in in Journal of the American Heart Association on November 28, 2023. You can access the article here.

**Fourth Trimester: Assessing Women's Health Equity and Long-Term Cardiovascular Outcomes in a Large Midwestern Health System in 2021**
Published in Circulation on November 27, 2023. You can access the article here.

**Million Hearts Cardiovascular Disease Risk Reduction Model.**
Published in Journal of the American Medical Association on October 17, 2023. To read, click here.

**Perspectives of African American Church Leaders in Response to COVID-19 Emergency Preparedness and Risk Communication Efforts Within a Community Engaged Research Partnership: COVID-19 emergency risk communication.**
Published in Disaster Medicine and Public Health Preparedness on October 13, 2023. You can access the article here.

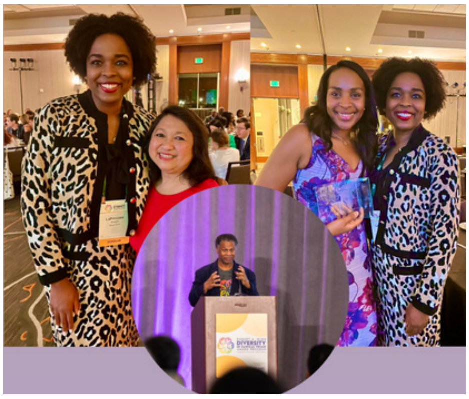

Dr. Brewer was a recipient of the Robert A. Winn Diversity in Clinical Trials Career Development Award, an award established by the Bristol Myers Squibb Foundation. This two-year program aims to train, develop and mentor diverse and community-oriented researchers and physicians to help increase the diversity of patients enrolled in clinical trials, and ultimately to enhance the development of therapeutics for all populations.

In November 2023, Dr. Brewer attended the Annual Convening of Robert A. Winn Diversity in Clinical Trials Conference in La Jolla, CA. Dr. Brewer was able to attend sessions related to clinical trial diversity.

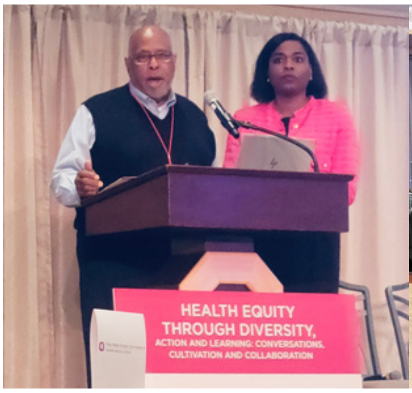


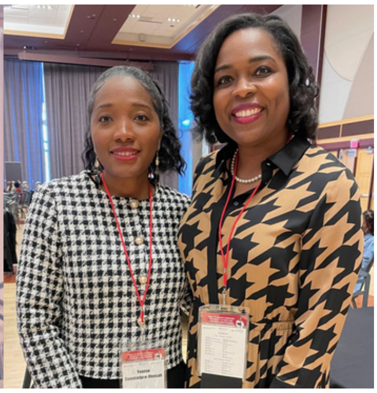

On October 13-14, 2023, Dr. Brewer and Clarence Jones co-presented at the Ohio State University Health Equity through Diversity, Action, and Learning Conference. This conference was co-chaired by Ohio State University, Ohio State University Public Health, and the City of Columbus Public Health.

To learn more about the conference, visit the conference website at: [https://www.hetdconference.com](https://www.hetdconference.com)

## Recent Outreach Events

### CPR & AED Training

On August 20, 2023, we were honored to partner with the American Heart Association and Minnesota Sudden Cardiac Arrest Survivor Network to increase CPR & AED training in the African American community. Many thanks to the Dan Abraham Healthy Living Center in Rochester, MN for hosting us! #HealthEquity #CPRSavesLives #3forHeart #LoveForDamar

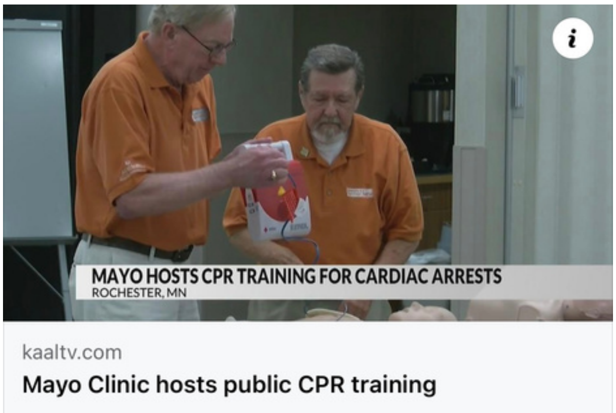


To view the news story, click here.

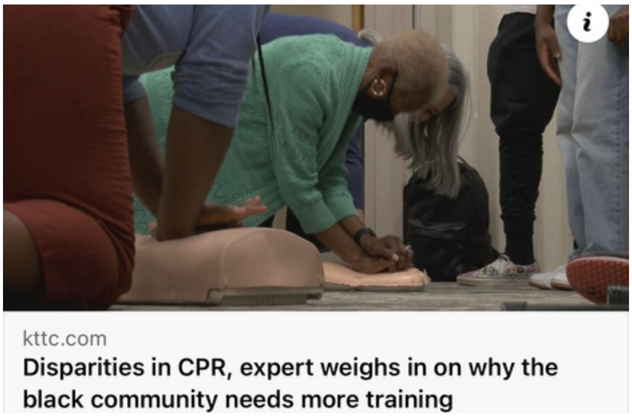


To view the KTTC news story, click here.

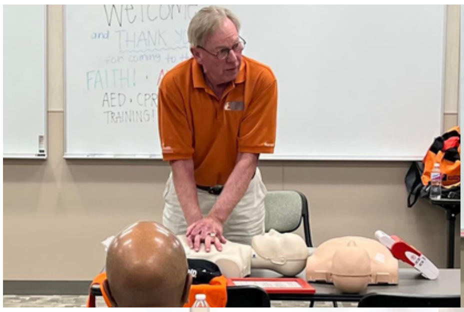

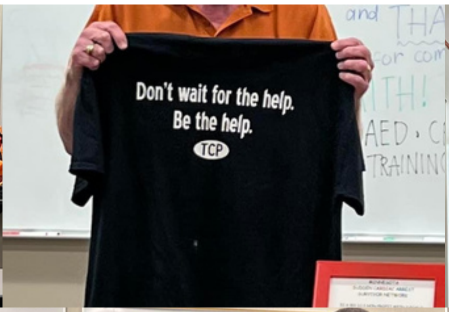


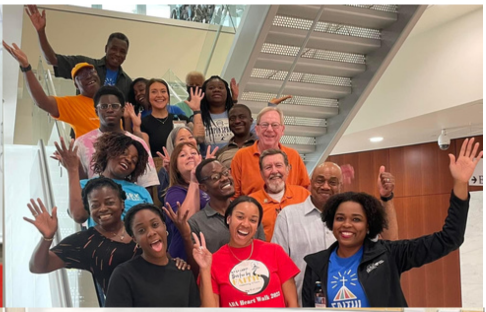

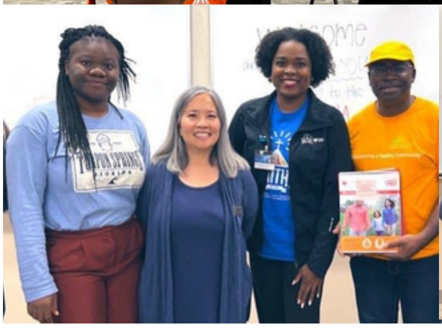

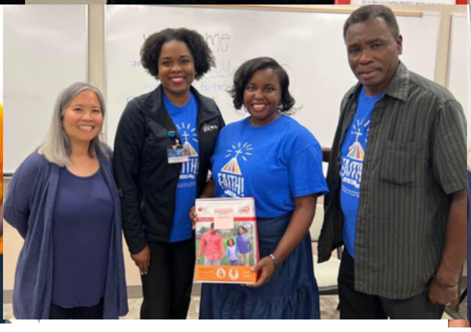

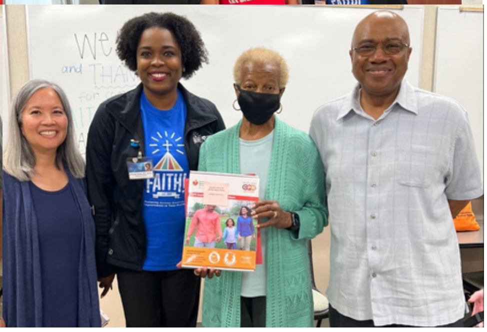

**Celebrating Dr. Brewer's Academic Promotion**

In October 2023, Christ's Church of the Jesus Hour, one of our Rochester church partners held a celebration in honor of Dr. Brewer and her promotion to Associate Professor of Medicine.

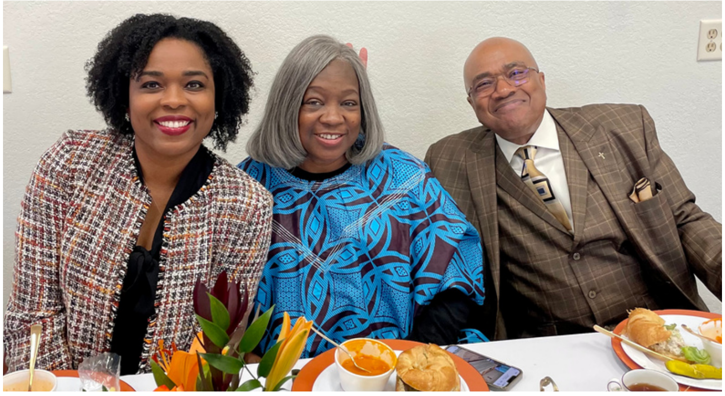

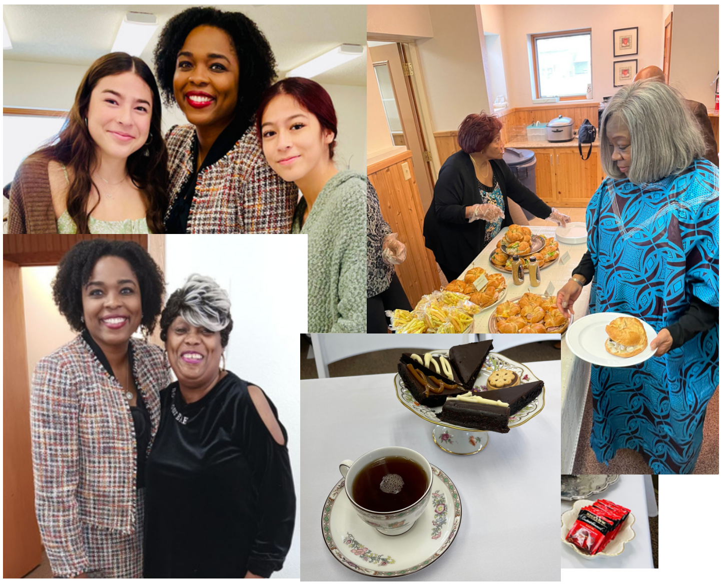

**FAITH! Community Steering Committee End of the Year Meeting**

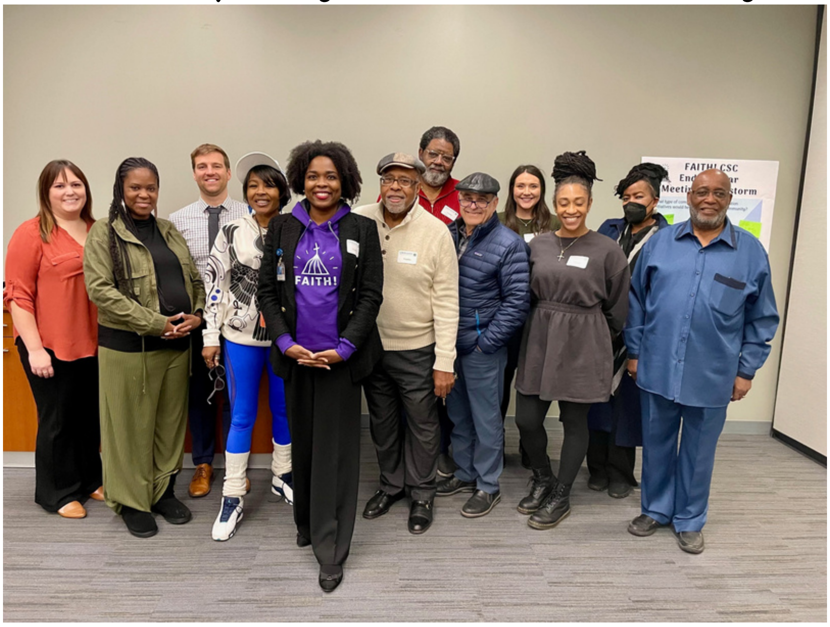


On December 8, 2023, we held our FAITH! Community Steering Committee (CSC) End of the Year Meeting at LifeSource in Minneapolis, MN. Lunch was catered by Soul Bowl, a black-owned restaurant. This was a hybrid meeting and allowed committee members to attend virtually. The meeting agenda consisted of CSC member updates, Committee updates, review of CBPR principles, and FAITH! Program updates. We also discussed potential new community outreach/education initiatives that we could integrate into our community engagement that would be most beneficial to the African American communities in Minneapolis and Rochester.

Keep an eye out for the next CSC meeting!

## BREWER COLLABORATORY NEWS

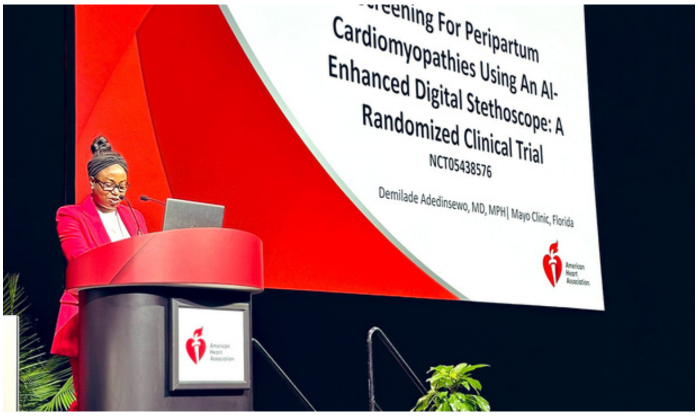


**Dr. Demi Adedinsewo, Mayo Clinic Florida Cardiologist**

In November 2023, Dr. Adedinsewo attended the 2023 American Heart Association (AHA) Scientific Meeting in Philadelphia, PA. As a part of the Late Breaking Session at AHA 2023, she discussed Artificial Intelligence for health equity and findings from the SPEC-AI Nigeria study.

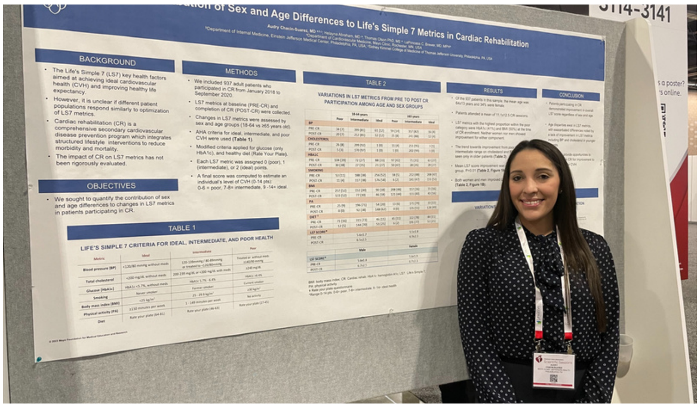


**Dr. Audry Chacin-Suarez, Jefferson-Einstein Internal Medicine Resident**

Dr. Audry Chacin-Suarez also attended the 2023 AHA Scientific Meeting. She was a poster presenter on "Contribution of Sex and Age Differences to Life's Simple 7 Metrics in Cardiac Rehabilitation".

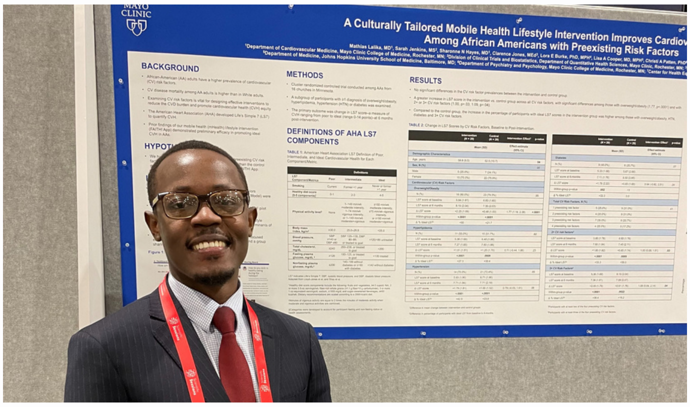


**Dr. Mathias Lalika, CHIEF Postdoctoral Research Fellow**
Dr. Lalika was a poster presenter at the 2023 AHA Scientific Meeting and presented our work, "A Culturally Tailored Health Lifestyle Intervention Improves CVH among AA with preexisting risk factors". Dr. Lalika also served as a judge for the Elizabeth Barrett-Connor Research Award for Early Career Investigators in Training Competition.

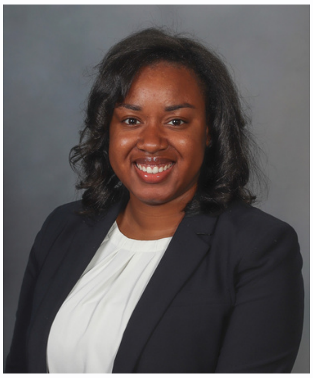

**Congratulations, Ashya on your acceptance into graduate school!**
Ashya Burgess was recently accepted into the Master of Medical Sciences program at Ponce Health Sciences University - St. Louis campus, and will begin classes in January 2024. After graduation, Ashya plans to re-apply to medical school.

Congratulations, Ashya!

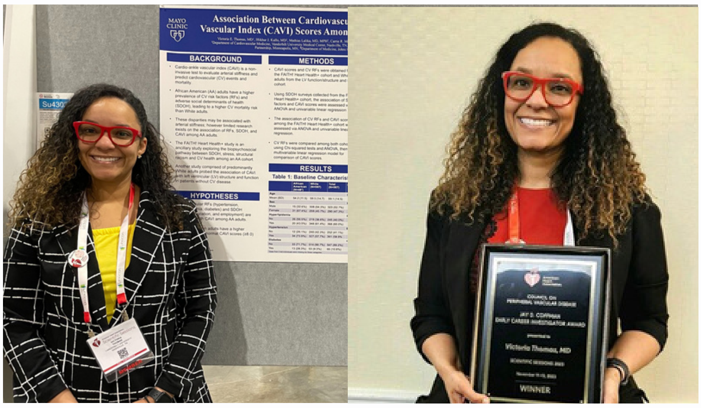


**Dr. Victoria Thomas, Vanderbilt University Cardiology Fellow**
Dr. Victoria Thomas presented her poster "Association Between Cardiovascular Risk Factors, Social Determinants of Health and Cardio-Ankle Vascular Index (CAVI) Scores Among African-American Adults: The FAITH! Heart Health+ (HH+) Study at the 2023 AHA Scientific Meeting.

Dr. Thomas was not only selected as a finalist of the 2023 AHA Research Council on Peripheral Vascular Disease Coffman Early Career Investigator competition, but she was also the award winner.

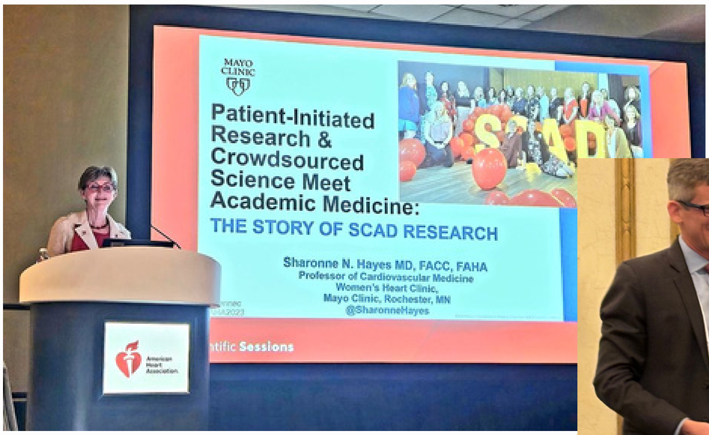


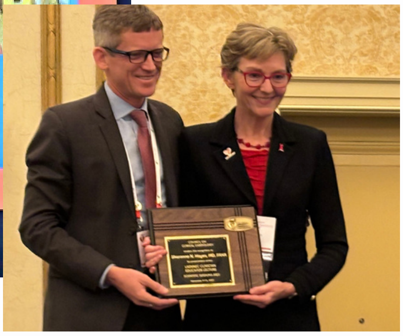

**FAITH! Collaborator Honored with AHA Award**
Congratulations to FAITH! collaborator, Dr. Sharonne Hayes who was honored with the prestigious American Heart Association Science Laennec Clinician-Educator Award from the Council on Clinical Cardiology at the 2023 AHA Scientific Meeting. She gave a superb lecture on "Patient-Initiated Research & Crowdsourced Science Meet Academic Medicine: The Story of SCAD Research".

**Brewer Collaboratory Team Outing**
Members of the Brewer Collaboratory Team were fortunate to gather this year at South x Southeast MN Brewing Company to connect and celebrate the accomplishments of 2023.

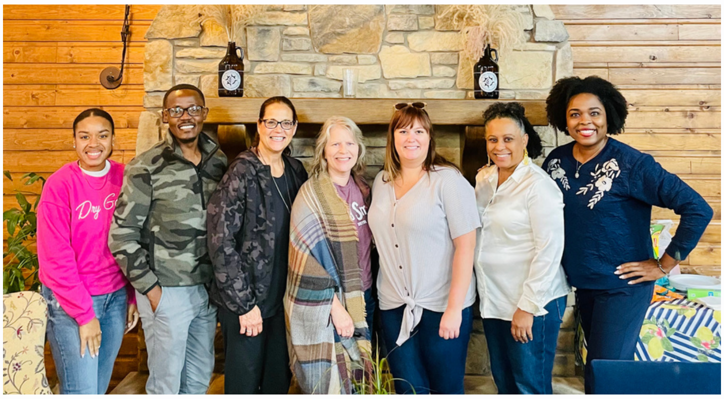

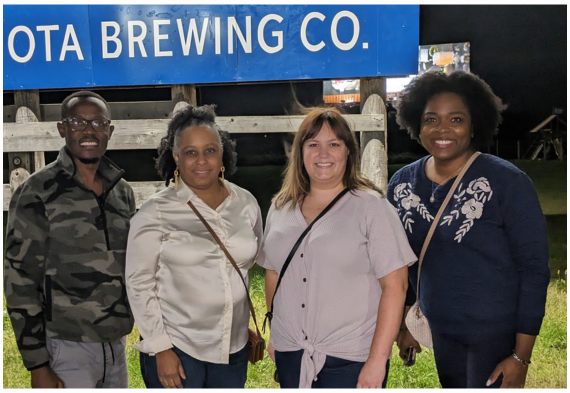

## CAREER, TRAINING & FUNDING OPPORTUNITIES

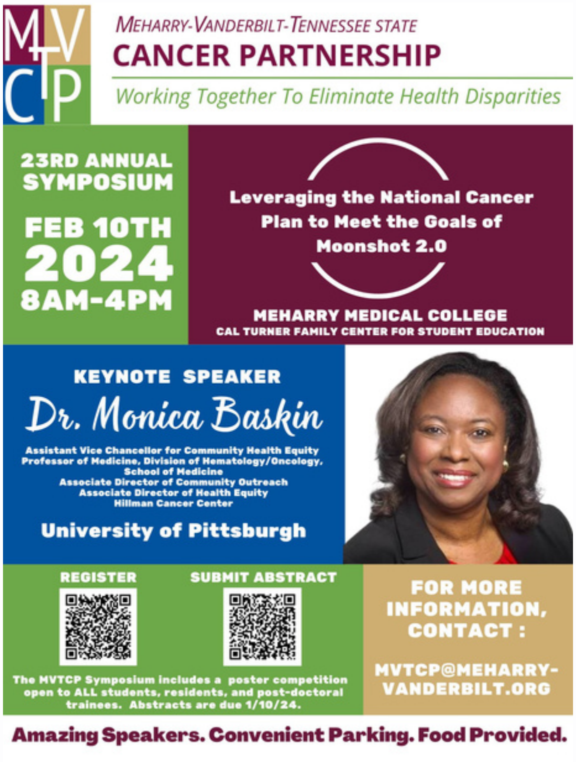


### 23rd Annual Meharry-Vanderbilt-Tennessee State Cancer Partnership Symposium

Date: February 10, 2024
Time: 8-4pm

To register for the conference, click here.

To submit an abstract, click here.

Abstract submission deadline: Jan. 10, 2024

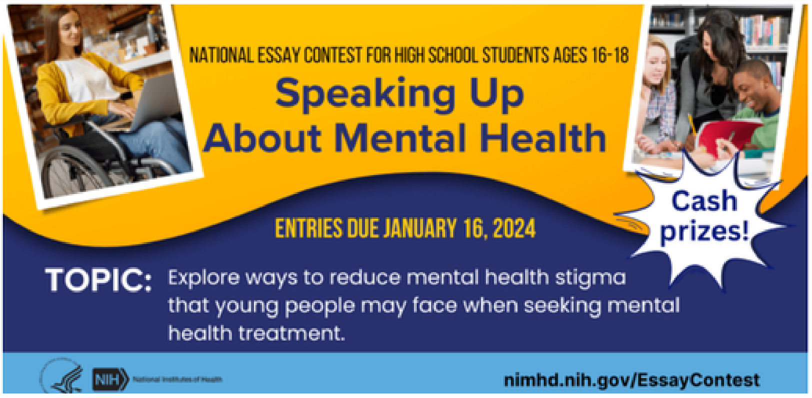


### NIH Mental Health Essay Contest for Teens

Find more contest details here.

You can read the winning essays from 2022 here.

Application deadline: Jan. 16, 2024

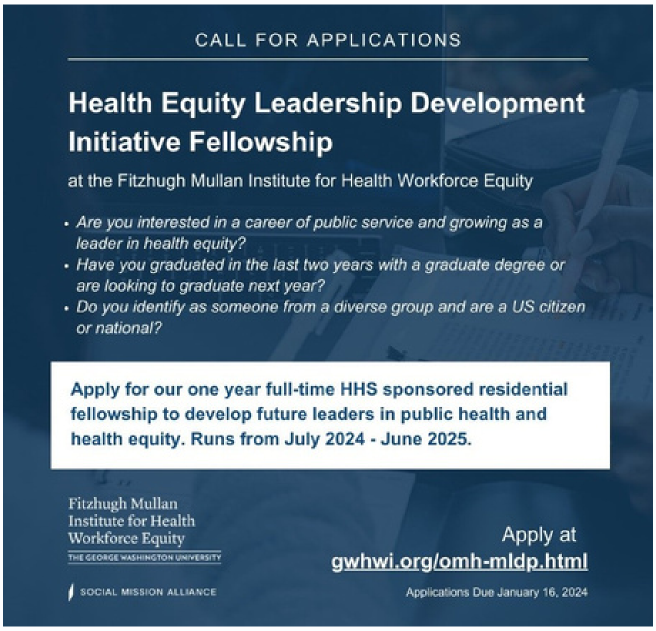


### The Office of Minority Health (OMH) Health Equity Leadership Development Initiative (HELDI) at the Mullan Institute

The OMH HELDI at the Mullan Institute aims to develop and support future federal leaders in public health and health equity. The program is sponsored by the Department of Health and Human Services and led in partnership with the Office of Minority Health.

The OMH HELDI is a one-year full-time residential fellowship program in the Washington, D.C. area. Fellows will receive a stipend for the fellowship year of July 2024 - June 2025.

Application deadline: Jan. 16, 2024

Learn more and apply at https://www.gwhwi.org/omh-mldp.html.

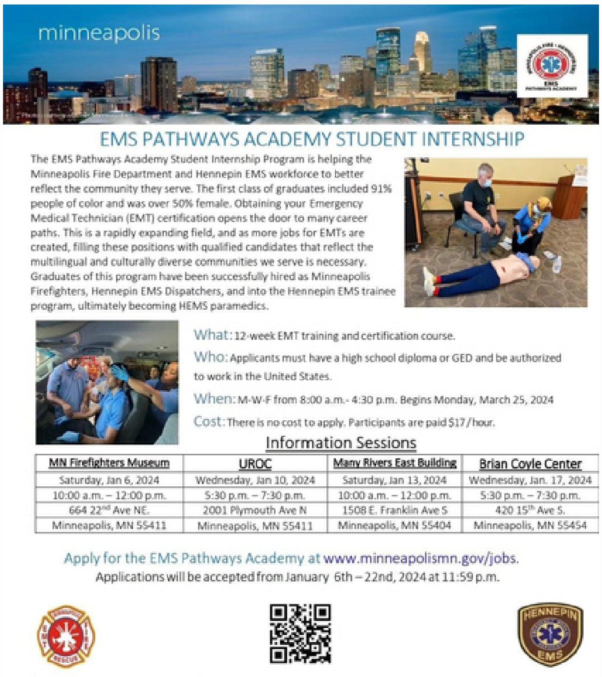


## EMS Pathways Academy Student Internship

To learn more and apply, click here:

Application deadline: Jan. 22, 2024

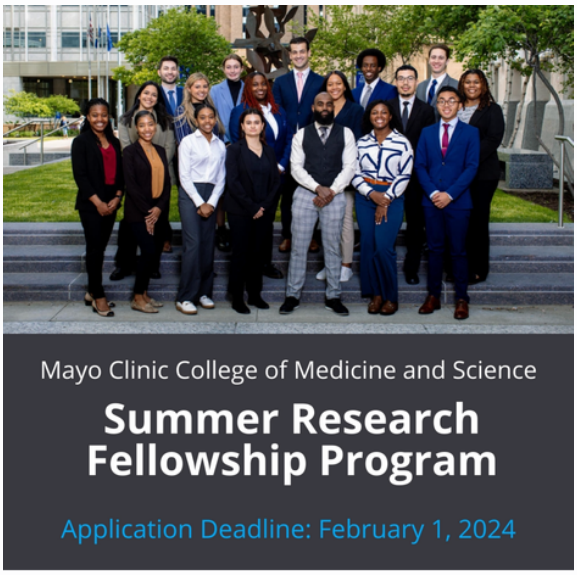


## Mayo Clinic College of Medicine and Science Summer Research Fellowship Program

The Summer Research Fellowship at Mayo Clinic is an 8-week comprehensive program open to underrepresented medical students.

To learn more and apply, click here.

Application deadline: Feb. 1, 2024

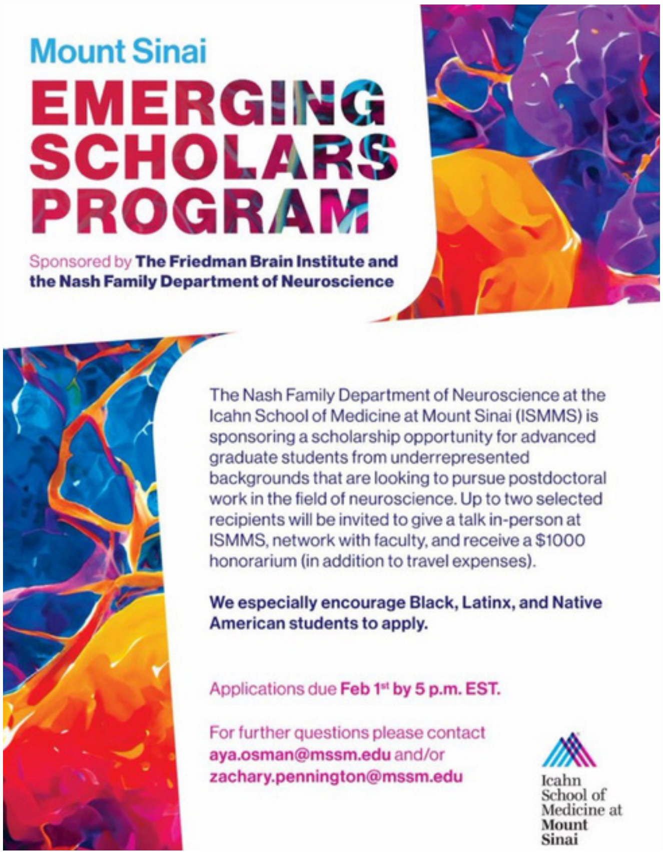


## Mount Sinai Emerging Scholars Program

Are you a neuro graduate student who wants a chance to give an invited talk at Mount Sinai, network with faculty and win $1,000?

To learn more and apply, click here.

Application deadline: Feb. 1, 2024

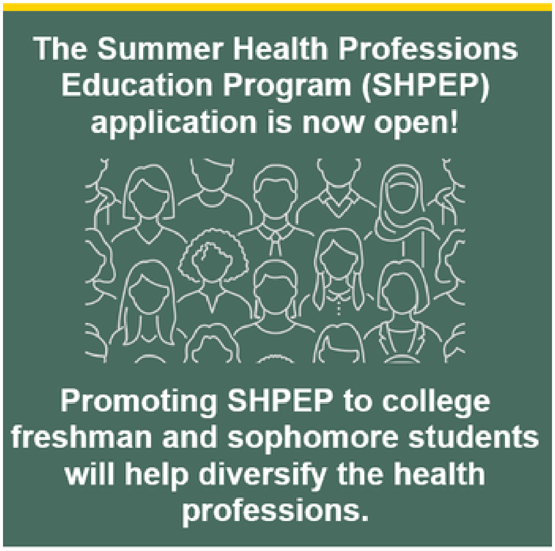


**SHPEP - Inspire college students to pursue a career in the health professions**

The Summer Health Professions Education Program (SHPEP) is a FREE transformative six-week summer experience for students interested in the health professions. SHPEP scholars have a legacy of success: over 65% of scholars who apply to medical or dental school are accepted.

To learn more and apply, click here:

Application deadline: February 5, 2024

**MN Department of Health Center for Health Equity**

Health equity champions and community partners are invited to subscribe to Health Equity Funding Opportunities. Click here to subscribe.

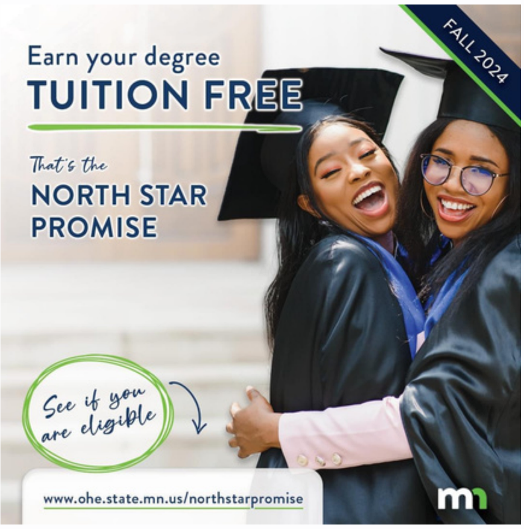


**North Star Promise Scholarship Program**

Starting in Fall 2024, the new North Star Scholarship Promise Program will cover the FULL COST of tuition and fees for students attending a MN public college w/ an annual income of <80k.

Application Deadline: TBA

Click here to learn more.

## HEALTH LITERACY

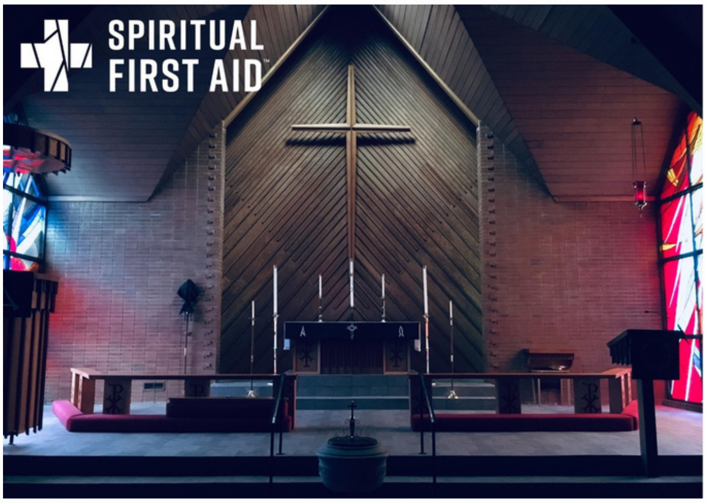


**Get Certified in Spiritual First Aid**

Burnout is at an all-time high across the U.S. This includes Pastors & those serving in ministry in various capacities.

We believe there's no Spiritual Aid without self-aid, which is why we're committed to giving you the tools you need to help you prevent, address, and overcome burnout. Learn more by clicking here.

Register today for FREE (originally $129) by emailing faith4heart@mayo.edu

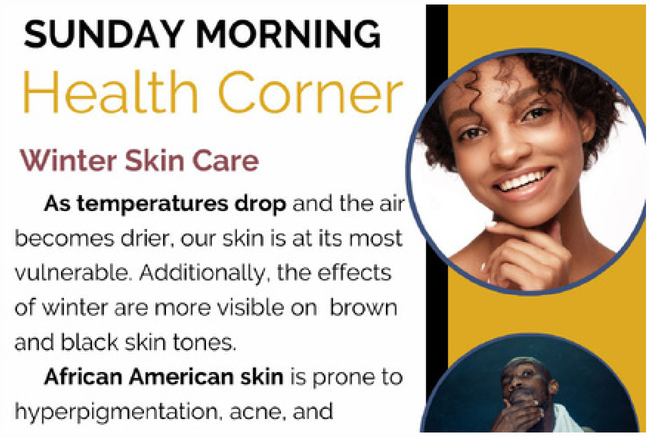


### The Balm in Gilead Inc.

Sunday Morning Health Corner is your congregation's online Health Resource Center to keep your congregation and community educated and informed about health issues and concerns related to the African American community.

Check out the Winter Skin Care resource provided by The Balm in Gilead Inc. by clicking here.

### Facts & Faith Fridays: Bridging faith and science in the community

Facts & Faith Fridays is a partnership between VCU Massey Comprehensive Cancer Center and the African American faith-based community. Led by community leaders, Rudene Mercer Haynes, Rev. F. Todd Gray, and Robert A. Winn, M.D., the program was founded in response to the COVID-19 pandemic. Facts & Faith Fridays recognizes that faith leaders are trusted sources of accurate information and act as ambassadors for their communities.

Massey invites you to join national experts and community leaders for a monthly conversation around science and religion as we work to enhance the lives of people in our communities.

Date: December 15, 2023
Topic: "Pancreatic Cancer & Year in Review Celebration with Dr. Luni Emdad and the Facts & Faith Friday Team"
To register, click here: Meeting Registration - Zoom

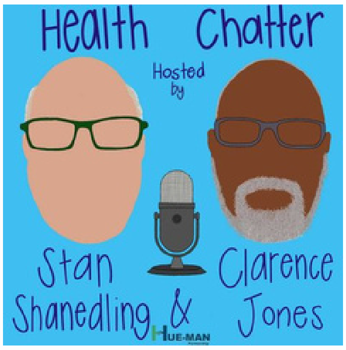


### Health Chatter Podcast

Dr. Stan and Clarence Jones partnered together and launched a podcast named "Health Chatter", a dedicated space to listen and share topics central to today's health.

Click here to learn more and tune in today.

## FRIENDS OF FAITH! ANNOUNCEMENTS

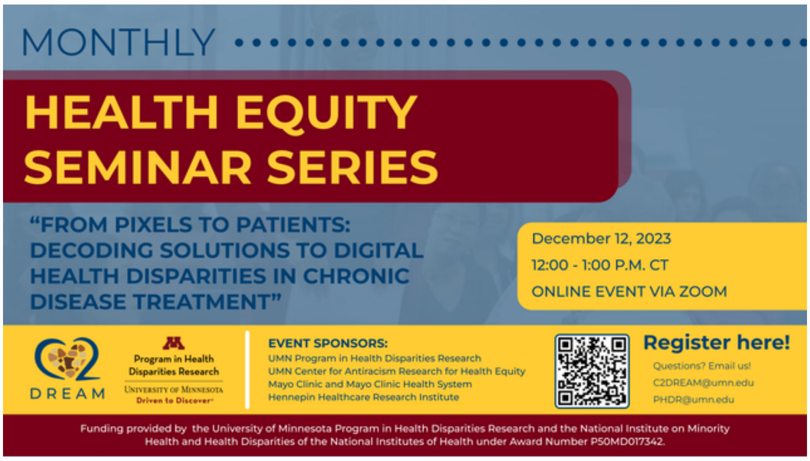


### Health Equity Seminar Series

"From Pixels to Patients: Decoding Solutions to Digital Health Disparities in Chronic Disease Treatment"

Presented by: Pravesh Sharma, MD

Date: December 12, 2023
Time: 12:00 - 1:00 PM CST
To register, click here.

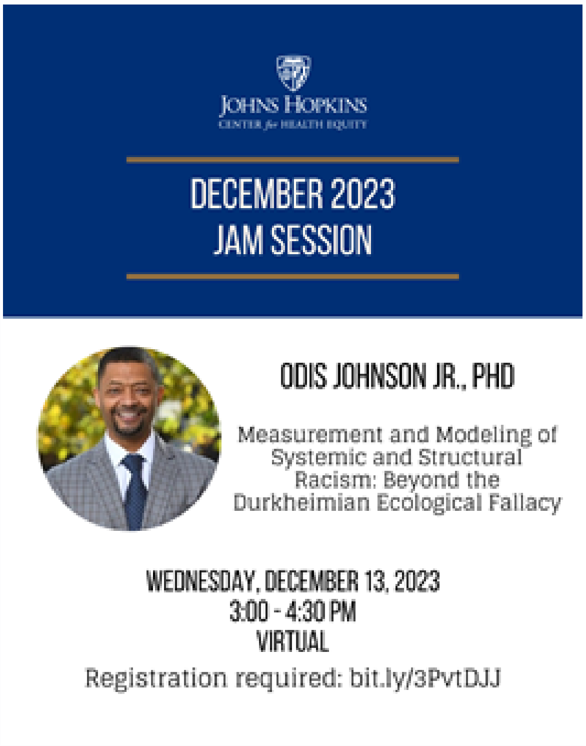


**Johns Hopkins December 2023 Jam Session**

"Measurement and Modeling of Systemic and Structural Racism: Beyond the Durkheimian Ecological Fallacy"

Date: December 13, 2023
Time: 3-4:30p ET
Virtual

To register, click here.

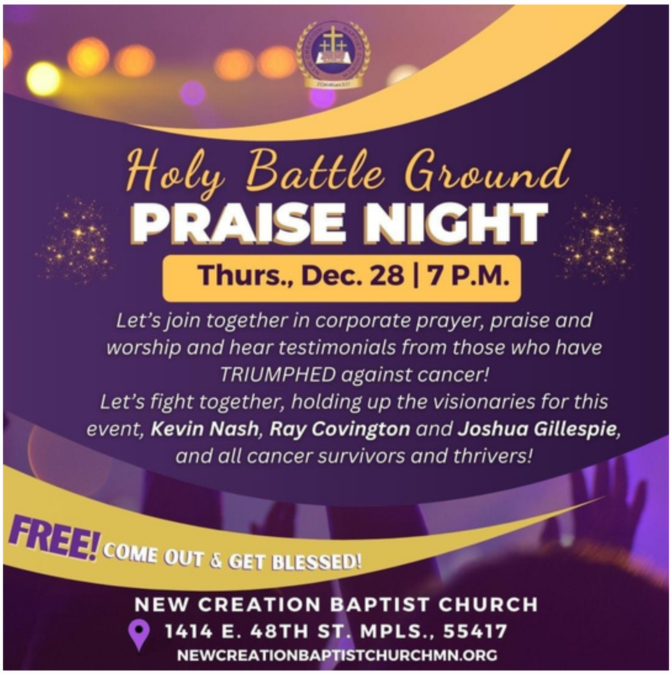


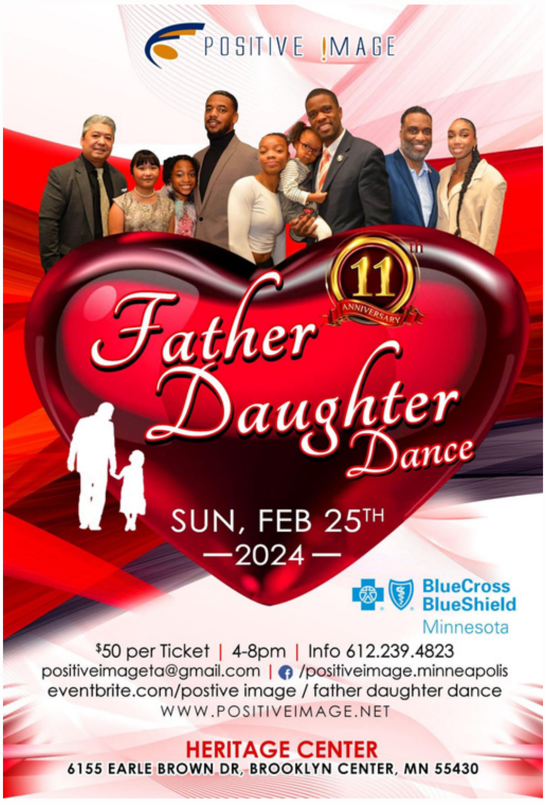


## SEASON'S GREETINGS

**The FAITH! team wishes you a happy holiday and happy new year!**

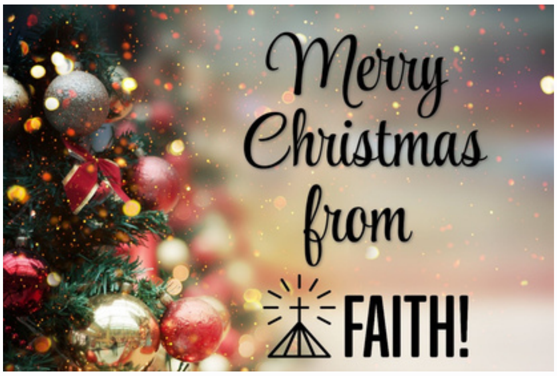


As we reflect on the past year, we are once again overcome with gratitude for your unwavering support as well as eager anticipation for a new year filled with learning growth, love, and faith.

May your days be filled with peace and happiness!

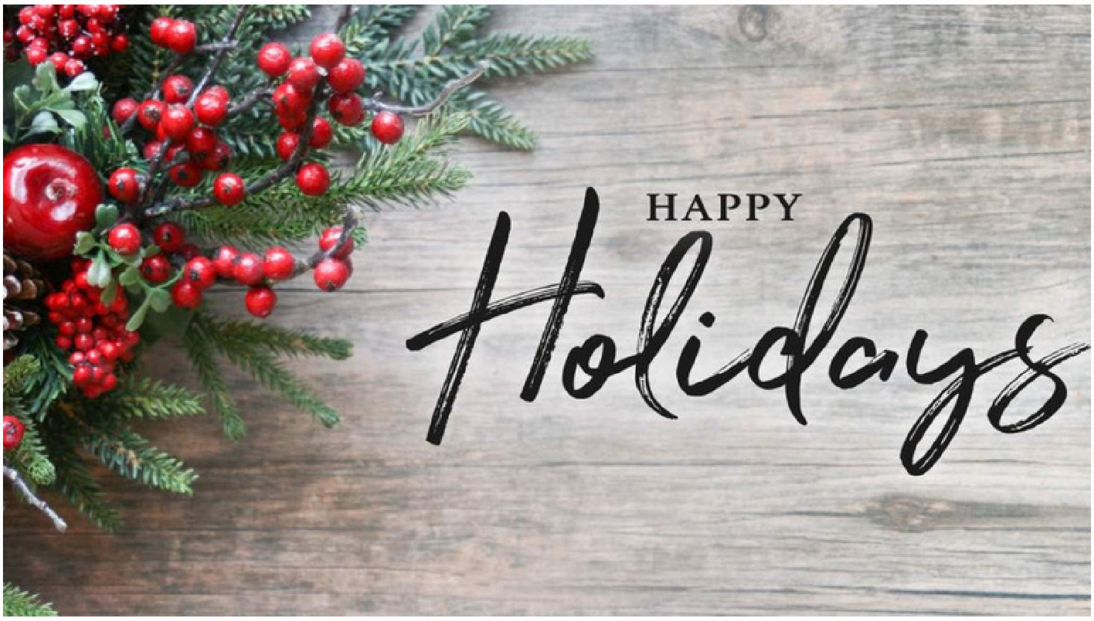


---

## CSC Birthdays

12/7 — Jacqueline Johnson

1/31 — Dr. LaPrincess Brewer

3/4 — Dr. Stan Shanedling

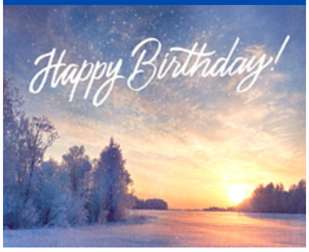


## Learn more about FAITH!

**FAITH! Promotional Video**

Click here to watch.

## Connect with us:

Follow us on FB @ FAITH4Heart!

Follow us on Twitter @ FAITH4Heart!

Email us @ FAITH4Heart@mayo.edu

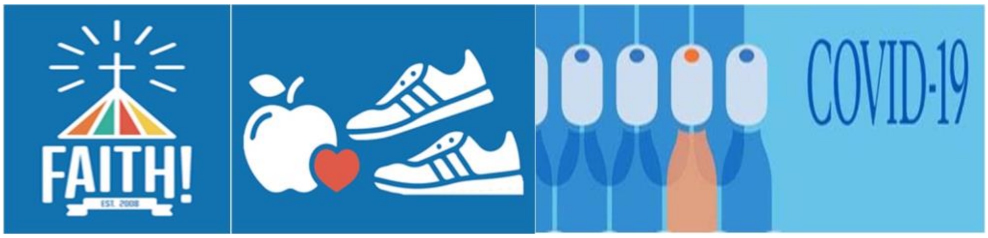


## Greetings FAITH! Community!

We hope you had a wonderful week. We hope you share this week's email with your congregation and find the resources to be helpful.

## SCRIPTURE OF THE WEEK

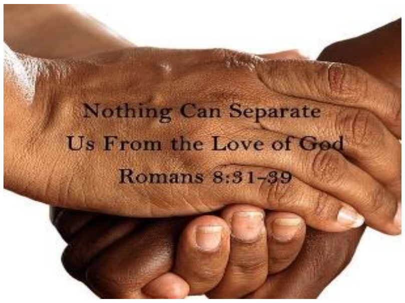


“Nothing can separate us from the love of God”. Romans 8:31

No matter what you feel you’ve done wrong or bad God will be ALWAYS love you for you!

## FAITH! Churches Spotlight: New Creation Baptist Church

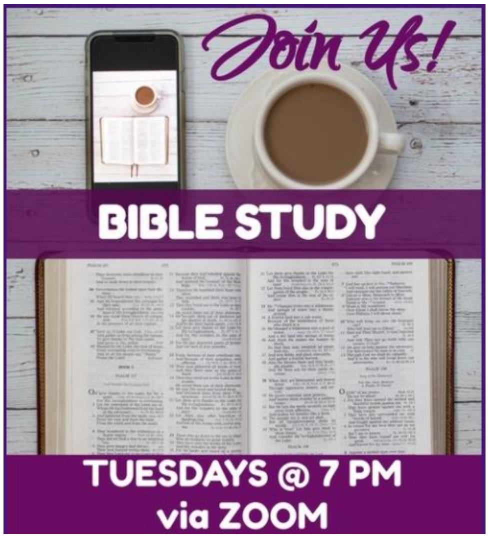


Join with other prayer warriors via Zoom Meeting/Call on Tuesdays at 7PM. For an invite, email newcreation1414@gmail.com.

For more information on this Bible Study, as well as other offerings, visit https://newcreationbaptistchurchmn.org/

## COVID-19 Emergency Preparedness Initiative! We are ready!

Thank you to all the churches that are now signed up for our exciting program to help our churches STAY READY!! And thank you to all the pastors who have completed their interviews. We are currently assessing each church's emergency preparedness and putting together personalized Emergency Preparedness binders!

Participating Churches:

1.) Creations Ministries COGIC
2.) Refuge International Ministries
3.) Riverside Evangelical Free Church
4.) New Hope Baptist Church
5.) New Creations Baptist Church
6.) Greater Friendship Missionary Baptist Church
7.) Full Proof Ministries COGIC
8.) Fellowship Missionary Baptist Church
9.) Word of Life Church of God in Christ
10.) St. James AMEC Church
11.) The Redeemed Christian Church of God Chosen Generation Parish
12.) Vision Church
13.) Christ's Church of the Jesus Hour
14.) New Oil Center COGIC
15.) St. Albans COGIC
16.) Immanuel Baptist Tabernacle Church
17.) Morning Star Church
18.) Great is Thy Faithfulness
19.) High Praise Ministries
20.) True Foundation Church of God and Christ
21.)

**Church videos (or photos) and short synopsis of how participating churches have used the FAITH! webinar series, manual, and incentives to enhance their emergency preparedness is due next month!!**

If any participating churches have any questions or concerns, please reach out to us at Faith4heart@mayo.edu!

The Emergency Preparedness Showcase will take place in January and will highlight each church and their individual Emergency Preparedness plans, initiatives, and more!

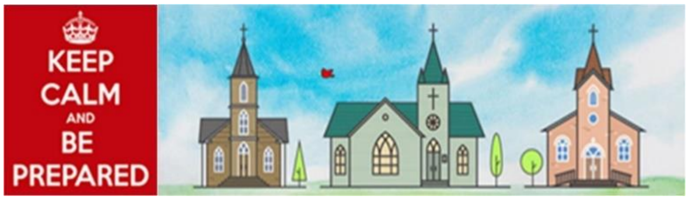

## FAITH! Emergency Preparedness Webinar Series

As part of our FAITH! Emergency Preparedness, we hosted a webinar series comprised of guest speakers who are experts in the field. If you missed any of our webinars, here they are in the order that we had them:

### Orientation: Saturday, April 10th

This provided an overview of the initiative is here: http://www.kaltura.com/tiny/vdp3k

### Seminar #1: Saturday, May 22nd

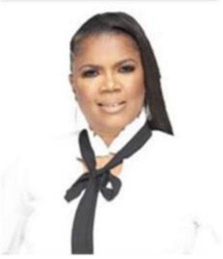

Featured the incomparable **Bishop La'Tresa Jester** of Gideon Baptist Church in Tucson, AZ! The topic of her presentation was Readiness is a Lifestyle – Phases of Disaster. The link to the recording of the seminar is here:
http://www.kaltura.com/tiny/z4c14

### Seminar #2, Saturday, June 12th

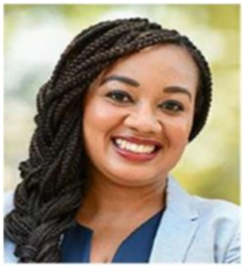

With guest speaker, **Dr. Nicolette Louissaint** of Healthcare Ready. Presentation topic was Building Resilient Churches. The recorded link is here:
**http://www.kaltura.com/tiny/suydb**

### Seminar #3: Saturday July 10th

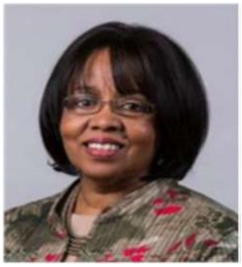

Guest speaker **Joyce Moon Howard**, DrPH, MPH, Clinical Associate Professor of Community Health Science and Practice, NYU School of Global Public Health. Topic: Partnerships for Preparedness, from Hurricanes to Pandemics. Here is the recorded link: http://www.kaltura.com/tiny/mwijk

### Webinar #4: Saturday, August 28th

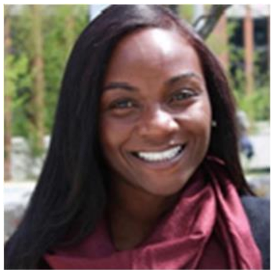

Guest speaker **Dr. Kizzmekia Corbett**, immunologist, assistant professor in the Department of Immunology and Infectious Diseases at Harvard T.H. Chan School of Public Health, and lead NIH scientist behind the development of the Moderna vaccine. Topic: (MIS)trust in the COVID-19 Vaccine in the African American Community.

https://newsnetwork.mayoclinic.org/discussion/virtual-public-forum-mistrust-in-the-covid-19-vaccine-in-the-african-american-community/

## Highlights of the week:

***Please send us information about what your church is doing to help our communities during this challenging time! We see you! We will share your information!**

### FAITH! Trial: Intervention Completed!

**Fantastic job to the FAITH! Trial participants! You have completed the 10-week intervention! REMEMBER!!!** You can and should continue to catch up on any missed or late modules, **and continue tracking on the app**, even though the 10 weeks are over! The tracking will continue until May 2022!!!

STEPS TAKEN:

3,312,876

FRUITS AND VEGETABLES TRACKED:

1,726

### Attention All FAITH Churches!!

We are reminding you that the Spiritual First Aid Training is still available for the church Pastor and Prevention Champion to do. If you or your champion has not received the code and the link for the online training, or the manual please email us so that we can help you with this as soon as possible.

## Community Spotlight: Culturally Responsive Caregiver and Dementia Services

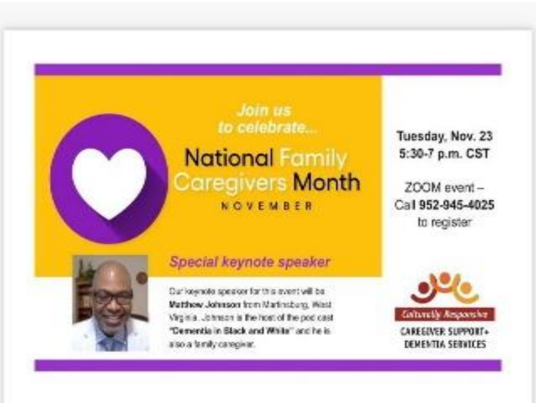


Please join the Volunteers of America for the National Family Caregivers Month celebration, Tuesday, November 23 from 5:30 to 7pm CST via zoom. To register or for more information call 952-945-4025.

For more information, follow https://www.voamnwi.org/culturally-responsive-caregiver-support-and-dementia-services

## Weekly Health Wellness and Good Readings updates:

### A tribute to world diabetes day!

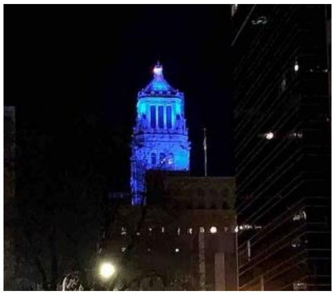

Mayo Clinic Buildings were lighted blue on November 14th in recognition of World Diabetes Day (WDD). WDD was created in 1991 by the International Diabetes Foundation and the World Health Organization in response to growing concerns about the escalating health threat posed by diabetes.
For more information about diabetes: https://mayocl.in/31QklBr

### 8 ways to eat healthy on a budget

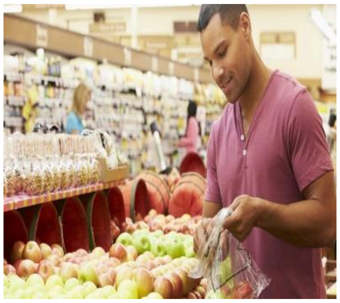

Eating healthy shouldn't break the bank — and it doesn't have to. You can make budget-friendly meals healthy and tasty with the right strategy.
Learn the tips and tricks for healthy shopping and meal-planning on a budget here: https://mayocl.in/30kzYAw

## Check with your doctor before getting vaccinated ladies!

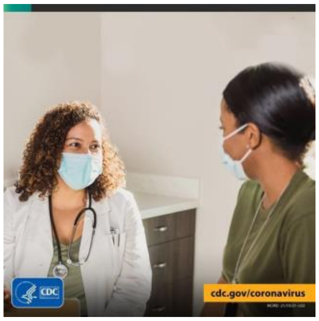


If you're due for a mammogram and have been recently vaccinated for COVID-19, ask your doctor how long you should wait to get your mammogram. This swelling is a normal sign that the body is building protection against COVID-19. However, it can cause a false reading on a mammogram. Learn more here: https://bit.ly/2YzXad7.

## Healthy Tip of the week: The Importance of Brain Health & Alzheimer's Awareness

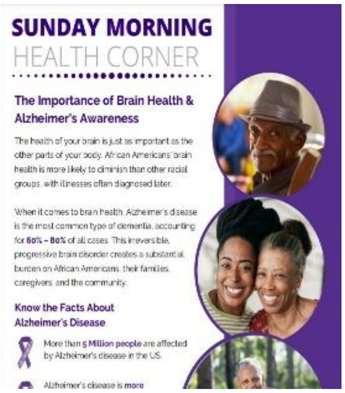


The health of your brain is just as important as the other parts of your body. African Americans' brain health is more likely to diminish than other racial groups, with illnesses often diagnosed later. Read the full pdf here: https://www.balmingilead.org/wp-content/uploads/2021/11/The-Importance-of-Brain-Health-and-Alzheimer_s-Awareness.pdf

## Tommy's Thanksgiving Weekend Presents: Don't shoot guns shoot hoops

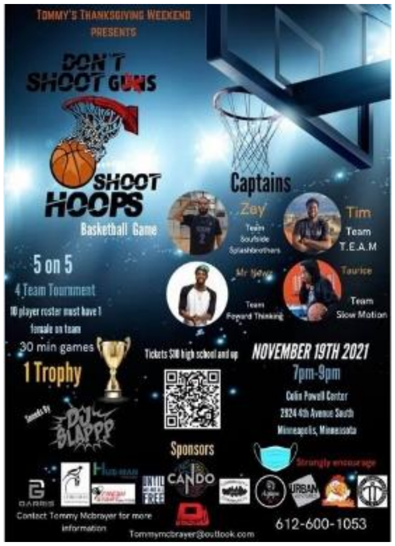


Come out to the 5 on 5 basket ball tournment to help stop the violience. Located at the Colin Powell Center 2924 4 ave south, minneapolis, minnesota on November 19, 2021 from 7PM-9PM. For more information on this event call TommyMcbrayer at 612-600-1053 or tommymcbrayer@outlook.com

## Dehydration signs to look for in kids!

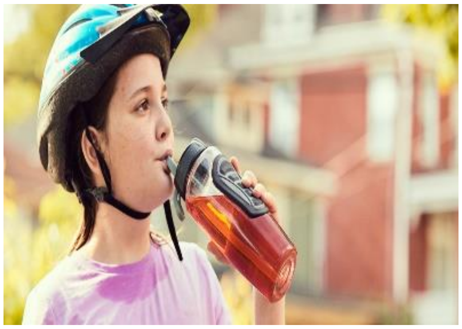

Drink eight glasses of water a day! We have all heard that advice time and time again. Hydration is part of what helps your body function properly.
But if you don't drink enough, you can become dehydrated. Dehydration can cause many issues, including headaches, dizziness, fatigue, fainting, rapid heartbeat, and other symptoms. Read more here: https://www.nutritionnews.abbott/nutrition-care/illness/dehydration-signs-in-kids/

## Facebook Page Information:

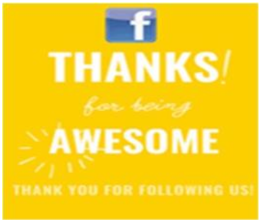


We have over **221** followers now **let's try to make it to 250!** Please continue to spread the word and encourage your friends, family, and congregation members to like and **follow our FAITH! Facebook page at FAITH! Cardiovascular Health & Wellness:**

**https://www.facebook.com/FAITH4Heart/**

We hope you find this information helpful. **Please feel free to contact us with any questions, concerns or needs.** We will do our best to provide you with the assistance you need. As you plan your weekend activities please remember to stay safe, practice social distancing and wear your mask.

Be Blessed, **The FAITH! Team**

Supplemental Document 3. 10 Commandments for a Healthy Heart During the COVID-19 Pandemic Flyer

# 10 Commandments
## FOR A HEALTHY HEART DURING THE
# COVID-19 Pandemic

Follow these steps to maintain a healthy lifestyle during the COVID-19 outbreak. Connect with Mayo Clinic for up-to-date information on heart health and COVID-19: www.MayoClinic.org

1
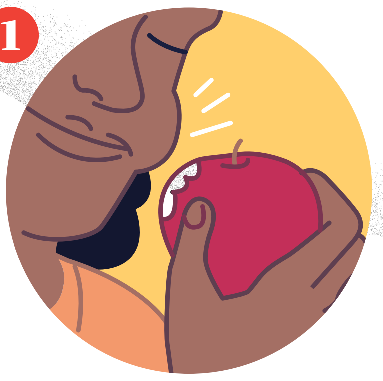

### Eat More!
Fruits and vegetables – at least 5 servings a day- and explore whole grains, plant proteins and healthy fats, such as olive oil.

2
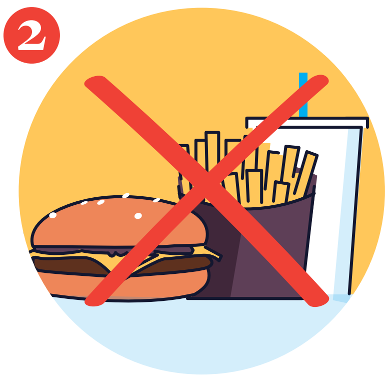

### Eat Less!
Limit processed and fried foods, foods and beverages with added sugars, high levels of sodium (salt) or saturated fat.

3
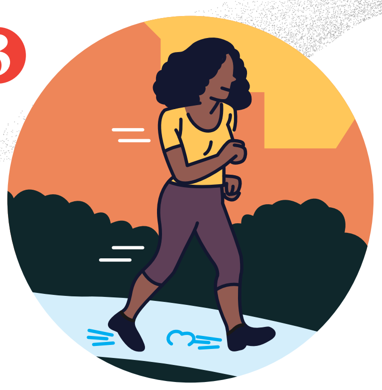

### Move More!
Try new fitness routines online that you can do within your home or walk/run while maintaining physical distance from others.

4
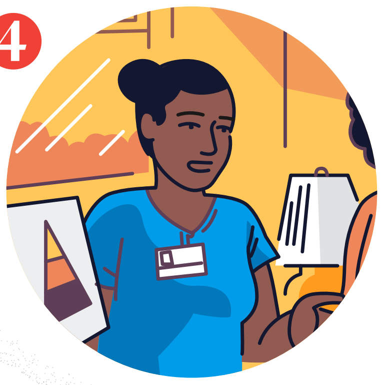

### Listen To Your Provider!
Follow recommendations, including medications and any special considerations related to your COVID-19 risk.

5
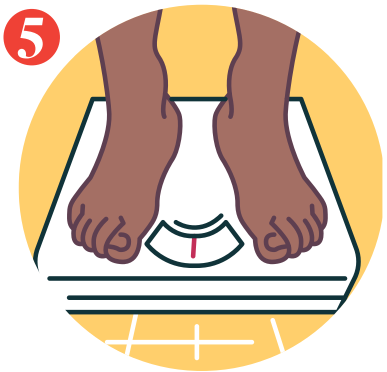

### Know Your Numbers!
Aim for a healthy blood pressure and weight. If you have diabetes, check your blood sugar regularly.

6
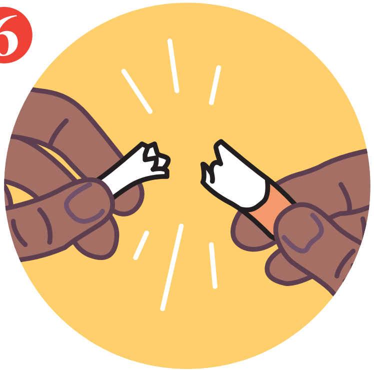

### No Smoking!
Do not smoke or use any other tobacco products!

7
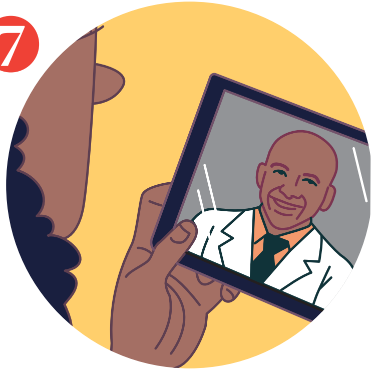

### Stay Connected!
Social distancing means “physical” distancing. Reach out to family members, friends and neighbors by phone or video chat.

8
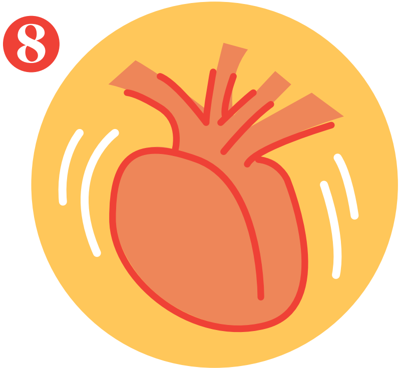

### Know Signs And Symptoms for Heart Attack or Stroke!
Seek medical attention immediately if you develop concerning symptoms.

9
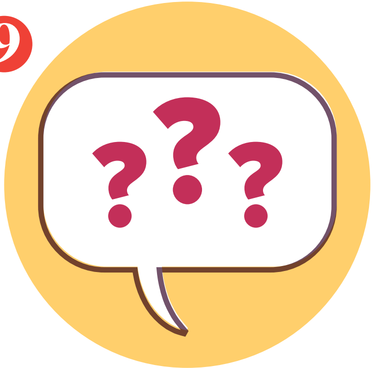

### Ask Questions!
Ask your healthcare provider for reliable information sources on COVID-19: mayoclinic.org/coronavirus-covid-19

10
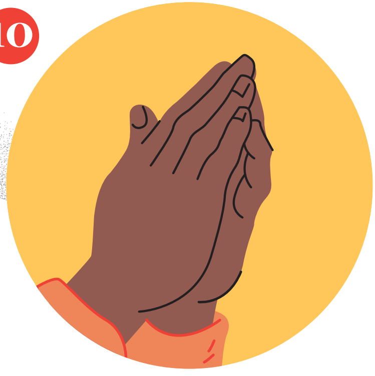

### Give Thanks!
We all have something to be grateful for despite this challenging time.

FAITH! EST. 2008

To learn more about the FAITH! project scan this QR Code with your smartphone!


**Contact Information**

LaPrincess C. Brewer, MD, MPH
Mayo Clinic Department of Cardiovascular Medicine

Phone: 507-538-0325

Email: brewer.laprincess@mayo.edu

@DrLaPrincess


©2020 Mayo Foundation for Medical Education and Research
